# Mechanochemical Activation of Olanzapine in Mixed Solid Dispersions: Impact of Excipients on Release and Permeation Rates

**DOI:** 10.3390/pharmaceutics18040411

**Published:** 2026-03-27

**Authors:** Tatyana Volkova, Olga Simonova, German Perlovich

**Affiliations:** G.A. Krestov Institute of Solution Chemistry RAS, 153045 Ivanovo, Russia; ors@isc-ras.ru (O.S.); glp@isc-ras.ru (G.P.)

**Keywords:** olanzapine, ternary solid dispersions, pharmaceutical excipients, permeation, dissolution rate

## Abstract

**Background**: The key parameters determining the bioavailability of an active pharmaceutical ingredient are its solubility/dissolution rate in physiological fluids and permeability across biological membranes. Highly accurate in vitro prediction of bioavailability is a key issue that typically arises during the development of new drug formulations and the improvement of existing ones. **Objectives**: The objective of the present work is to study the dissolution/release and permeation of olanzapine (OLZ) from two- and three-component solid dispersions (SDs) with sulfobutylether-β-cyclodextrin (SBE-β-CD) and several pharmaceutical adjuvants as solubilizing agents. **Methods**: Solid dispersions were prepared by mechanical grinding and characterized with X-ray Phase analysis (PXRD), Fourier Transform Infrared (FTIR) and Raman spectroscopy, Differential Scanning Calorimetry (DSC), and Scanning Electron Microscopy (SEM). **Results**: Raman spectroscopy was shown to be the best for revealing the interactions of OLZ with SBE-β-CD and γ-aminobutyric acid (GABA) in the three-component SD. The kinetic dependences of OLZ release and diffusion through the cellulose membrane were thoroughly described by quantitative parameters and classified according to the drug release mechanism. Significant improvement of release rate, OLZ concentration, and permeation with SDs compared to the pure OLZ was demonstrated. **Conclusions**: It was shown that the selected dispersions were stable when stored under normal conditions but underwent changes upon exposure to elevated temperature and humidity. The nature of these changes was determined by the properties of the components and their mutual interactions.

## 1. Introduction

The oral route of drug administration is one of the most common in clinical practice. The effectiveness of an oral active pharmaceutical ingredient (API) depends on its physicochemical properties and the physiology of the gastrointestinal (GI) tract. The most important parameters are stability, solubility, permeability across the intestinal epithelium, and transit time through the gastrointestinal tract segments where maximum absorption occurs. Most marketed drugs demonstrate poor solubility in aqueous biological fluids [1]. Over long periods of time, inadequate solubility/dissolution rate and bioavailability have remained significant challenges in small drug molecule development and require more complex delivery systems. Among other approaches based on various physical and chemical modifications [2], manipulation of the solid form of drug compounds through the formation of solid dispersions (SDs) containing various additional substances designated as pharmaceutical excipients is of paramount importance. Literature surveys from the past decade have demonstrated a considerable expansion in the range of excipients and their combinations used in the production of SDs [3]. The list of the most used excipients contains substances with hydrotropic properties (for example, urea, nicotinamide, sugars, such as dextrose, sorbitol, maltose, xylitol, mannitol, lactose, etc.), biopolymers (polyethylene glycols, polyvinylpyrrolidone, or cellulose derivatives such as hydroxypropylmethylcellulose), surfactants (polyoxyethylene and polyoxypropylene copolymers, polyoxyethylene lauryl ethers, polyethylene glycol glycerides, and others), and cyclodextrins. The advent of multi-component SDs has significantly enhanced the possibilities of modifying the dissolution properties of poorly soluble APIs. Among these, the ternary cyclodextrin-polymer SDs have been thoroughly investigated [4], whereas the information on polymer-hydrotropic substances or cyclodextrin-hydrotropic substances is rather scarce [5,6]. The studies in the mixed solutions containing polymer/hydrotrope or cyclodextrin/hydrotrope demonstrated the advantageous synergistic action of both excipients on the solubility of poorly soluble hydrophobic drugs [7,8]. One can expect the same effect on the dissolution and/or permeation of SDs based on hydrotrope mixtures with other excipients. An additional component in a solid dispersion may serve not only as a solubilizing agent but also as a modifier of both drug release and diffusion through the membranes. Oral dosage forms with modified release are of great clinical significance and are characterized by the greatest diversity [9]. A number of studies focusing on the solubility-permeability relationship of APIs in SDs is steadily increasing [10,11,12,13]. The benefits of SDs, such as the minimization of permeability reduction or even an increase in permeation rate, are well-documented [14].

The aspects of stability are of special importance, particularly for such compounds as olanzapine crystallizing in various crystal forms. The formation of different polymorphic forms, hydrates, and solvates [15] complicates the accurate identification of the solid state and brings complexities to the drug formulation process. The stability of a drug in SDs is influenced by several factors, such as drug-excipient interactions, drug loading, manufacturing process, and storage conditions. The selection of an appropriate excipient is crucial for the design of stable drug compositions. The incorporation of a third component to form ternary solid dispersions (TSDs) can enhance stability by forming hydrogen bonds with the drug and/or other excipients, which helps to stabilize the amorphous form of the drug and prevents its recrystallization [16]. In vitro dissolution testing is typically carried out to evaluate the performance of newly formulated solid dispersions. However, these experiments are insufficient for predicting the intestinal absorption. For this purpose, methods that can simultaneously examine the dissolution and permeation of an API released from a formulation are often employed [17] using a side-by-side Franz diffusion cell and various artificial and biomimetic membranes [18].

Olanzapine (OLZ) is an antipsychotic drug belonging to the thienobenzodiazepines class, structurally similar to clozapine, which is used for the treatment of schizophrenia and bipolar affective disorder [19]. It belongs to Class II of the Biopharmaceutical Classification System (BCS) and exhibits inherently high permeability through biological membranes, but low aqueous solubility. OLZ was previously formulated into various solid dispersions with different excipients, resulting in improved aqueous solubility and dissolution rate compared to the pure drug [20,21,22]. But no studies on mixed solid dispersions based on OLZ could be found in the recent literature.

**Figure 1:**
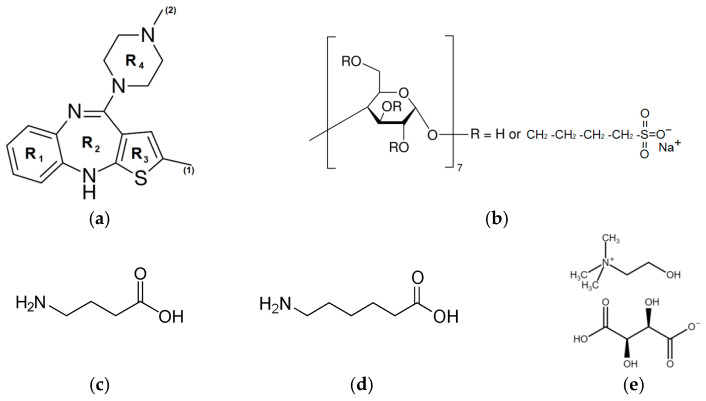
Structures of the studied compounds: (**a**) olanzapine (OLZ), (**b**) sulfobutylether-β-cyclodextrin (SBE-β-CD), (**c**) γ-aminobutyric acid (GABA), (**d**) 6-aminocaproic acid (6ACA), and (**e**) choline bitartrate (ChB).

The aim of the present study was to prepare the two-component (1:1) solid dispersions of OLZ with sulfobutylether-β-cyclodextrin (SBE-β-CD), γ-aminobutyric acid (GABA), 6-aminocaproic acid (6ACA), choline bitartrate (ChB), and three-component solid dispersions OLZ/SBE-β-CD/GABA (1:0.25:0.75) and OLZ/SBE-β-CD/GABA (1:0.75:0.25) by ball milling technique and to examine and quantitatively characterize the dissolution and permeation rate of olanzapine upon its release from SDs. To achieve the maximum effect and in accordance with the recommendations reported in the literature [23], the dissolution and permeability experiments were conducted in a buffer medium at pH 7.4, in which the compound exhibited minimal solubility. The prepared SDs were thoroughly characterized by X-ray Phase analysis (PXRD), Fourier Transform Infrared (FTIR) and Raman spectroscopy, Differential Scanning Calorimetry (DSC), Scanning Electron Microscopy (SEM) methods. The structures of OLZ and the excipients used are illustrated in Figure 1.

Due to its polyanionic nature, SBE-β-CD binds particularly well to cationic, nitrogen-containing compounds [24,25]. SBE-β-CD is easily soluble in water (>500 mg·mL^−1^), has proven its worth as a safe solubilizer and stabilizer, is well tolerated in humans, and does not cause adverse effects on the kidneys or other organs following either oral or intravenous administration [26].

Optimal concentrations of excipients in dosage forms are selected to impart the necessary physicochemical and biopharmaceutical properties and to ensure the product’s quality, stability, safety, and efficacy. In many cases, this can be achieved by using a combination of several excipients rather than an excessive amount of a single one, thereby reducing their individual toxicity. In this study, we used ChB, GABA, and 6ACA as additional components of SDs alongside SBE-β-CD.

The bioactive compound GABA, a non-proteinogenic amino acid widely used in foodstuffs and pharmaceuticals, occurs widely in microorganisms, plants, and vertebrates. It is well known that GABA is the primary inhibitory neurotransmitter in the central nervous system, suppresses neurotransmission and tranquilization, manifests hypotensive/antihypertensive and diuretic effects, and also helps prevent sleeplessness and depression [27]. As a hydrotropic agent, GABA can enhance the solubility of certain compounds in water, similar to other hydrotropes. The dual role of GABA as both a neurotransmitter and a hydrotropic agent justifies its use in pharmaceuticals. It is a polar molecule, highly soluble in water, and capable of forming hydrogen bonds. GABA can modulate the permeability of intestinal epithelial cells, potentially influencing gut–brain communication [28].

The hydrophobic flexible structure of the ω-amino acid (6ACA) allows it to be used as a linker in various molecules and to improve the solubility of hydrophobic compounds due to its hydrotropic potential [29]. This compound is approved by the FDA for use in the treatment of acute bleeding caused by elevated fibrinolytic activity [30].

Short-chain choline carboxylates ChC_m_ with m = 2, 4, 6 (including ChB) function as hydrotropes, solubilizing hydrophobic compounds in aqueous solution, whereas the longer-chain choline carboxylates ChC_m_ with m = 8, 10 and choline oleate are capable of forming micelles [31].

The solubilizing effect of SBE-β-CD and ChB on OLZ was confirmed in our previous study [8]. Amino acids were utilized for their hydrotropic potential towards hydrophobic drug substances. Special attention was paid to GABA due to its antidepressant activity. Its combination with OLZ could serve as a basis for new pharmaceutical formulations.

## 2. Materials and Methods

### 2.1. Materials

Information on olanzapine and the chemicals used, including their main identification characteristics and sources, is provided in Table S1 (Supplementary Information). All chemicals were used as received. The structures of the investigated compounds are presented in Figure 1.

### 2.2. Buffers Preparation

Bidistilled water (2.1 μS cm^−1^ electrical conductivity) was used to prepare the buffer solutions. Phosphate buffer pH 7.4 (I = 0.26 mol∙L^−1^) was prepared from KH_2_PO_4_ (9.1 g in 1 L) and Na_2_HPO_4_∙12H_2_O (23.6 g in 1 L) salts. The pH values of the solutions were monitored using an FG2-Kit pH meter (Mettler Toledo, Greifensee, Switzerland), standardized with pH 4.00 and 7.00 solutions. The pH was adjusted with 1 M NaOH or 1 M HCl solutions.

### 2.3. Preparation of Two- and Three-Component Solid Dispersions

The following two- and three-component solid dispersions (SDs) were fabricated by mechanical grinding using a planetary ball micro mill Fritsch Pulverisette 7 (Burladingen, Germany): OLZ/ChB (1:1)-SD, OLZ/SBE-β-CD (1:1)-SD, OLZ/GABA (1:1)-SD, OLZ/6ACA (1:1)-SD, OLZ/SBE-β-CD/GABA (1:0.25:0.75)-SD, and OLZ/SBE-β-CD/GABA (1:0.75:0.25)-SD. The molar ratios of the components are given in parentheses. The respective physical mixtures (pm) were prepared by accurately weighing and gently blending the components in the indicated molar ratios. A physical mixture sample (~50–60 mg) was placed into a 12 mL agate jar with twelve 5 mm agate balls and ground at 500 rpm for 1 h, with a 5 min pause introduced after 30 min to avoid overheating, as reported previously [8]. The solid dispersions (SDs) were collected and comprehensively characterized using PXRD, FTIR, Raman spectroscopy, DSC, and SEM. The samples were then sieved through a 150 μm sieve to obtain a particle size fraction of 80–150 μm. For further testing, the sieved fraction was stored in a desiccator at room temperature, protected from light. For comparison, and to evaluate the impact of grinding, raw OLZ was ground under the same conditions as the SDs.

Descriptions of the characterization methods, including PXRD, FTIR, Raman spectroscopy, DSC, and SEM, can be found in Supplementary Materials, Section S1.

### 2.4. Dissolution Measurements and Calculation of Quantitative Parameters

Dissolution/release experiments were conducted using a combined dissolution/permeation setup equipped with a side-by-side diffusion cell (PermeaGear.de H1C SIDE-BI-SIDE Diffusion System, SESGmbH-Analytical system, Bechenheim, Germany). Both donor and acceptor compartments have a capacity of 7 mL. Dissolution experiments were conducted under nonsink conditions according to the procedure described in the literature [32]. According to the authors, under nonsink conditions, the results more accurately predict the in vivo behavior of drugs for which dissolution is limited by solubility. This is ideal for testing amorphous solid dispersions and discriminating between formulations. A sheet of aluminum foil was mounted in place of the membrane to avoid the effect of changes in the donor concentration due to sampling and replacement with fresh buffer on permeability, as described elsewhere [33]. Samples of OLZ and the SDs were placed in a pH 7.4 aqueous buffer dissolution medium. The sample amount was determined based on the volume (250 mL) available in the intestinal lumen for dissolution of a single oral dose of OLZ (20 mg). The amount of SD was calculated separately for each sample based on the amount of OLZ equal to 0.56 mg in 7 mL. The experiments were conducted at a constant temperature of 37 °C (maintained with a circulating water bath) and a stirring speed of 500 rpm. The experiment duration was 7 h. Solution samples (0.4 mL) were collected at predetermined time points (5, 10, 20, 30, 45, 60, 90, 120, 180, 240, 300, 360, and 420 min) and replaced with an equal volume of fresh dissolution medium to maintain a constant total volume. After filtration through a 0.45 μm nylon syringe filter, sample concentrations were determined. The concentration of olanzapine was quantified using a validated UV-Vis spectrophotometric method (Varian Cary-50 spectrophotometer (Palo Alto, CA, USA, Software Version 3.00 (339))). The absorbance was measured at λ_max_ = 256 nm using 10 mm quartz cuvettes. The method was validated according to ICH guidelines and showed good linearity (R^2^ > 0.999) over the concentration range of 3.23–70.4 µM. The precision was RSD < 2.46%, and sensitivity LOD = 2.17 µM and LOQ = 6.58 µM were found to be satisfactory, with no interference from the excipients in the medium.

Based on the experimental results, the dissolution/release profiles were plotted. Quantitative parameters characterizing the curves were then calculated. The dissolution performance parameter quantifying the rate and extent of release from a dosage form was calculated as follows:

(1) $$DPP(\%)=\frac{{AUC}_{actual}}{{AUC}_{theoretical}}\times 100\%$$

where $AUC_{actual}$ and $AUC_{theoretical}$ is the integral area under the curve C_0_C_t_, and C_max_C_max_. To compare the dissolution profiles, the similarity factor (*f*_2_) was calculated with a pair-wise procedure [34]:

(2) $$f_{2}=50\times \log\left\{{\left[1+\left(\frac{1}{n}\right)\sum_{j=1}^{n}{\left|R_{j}-T_{j}\right|}^{2}\right]}^{-0.5}\times 100\right\}$$

where *n* is the sampling number, and *R* and *T* are the percent OLZ dissolved from the reference and test solid samples at each time point *j*.

To elucidate the release mechanism, the data were fitted to the Korsmeyer–Peppas power-law equation [35]:

(3) $$\frac{Q_{t}}{Q_{\infty }}=k\times t^{n}$$

In this equation, *Q*_*t*_/*Q*_∞_ is the fractional release of the drug at time *t*, *k* is the release constant, which reflects the structural modifications and geometrical characteristics of the system, and *n* is the diffusional exponent that indicates the release mechanism. In brief, an *n* value ≤ 0.5 indicates a Fickian diffusion mechanism, suggesting that drug release is primarily driven by the concentration gradient. When 0.5 < *n* < 1.0, it signifies non-Fickian anomalous transport governed by a combination of diffusion and matrix swelling/erosion [36]. When *n* is approximately 1, the release follows zero-order kinetics (i.e., the release rate is constant over time). This mechanism, known as Case II transport, is driven by the swelling and relaxation of the polymer chains. An *n* > 1.0 denotes super Case II transport, where drug release is primarily governed by polymer erosion. The initial 60% of the fractional release (a range less affected by drug depletion and matrix alterations) was selected for model fitting with Equation (3) [37].

### 2.5. Permeation Experiments

Permeation/diffusion rates were measured in a side-by-side diffusion cell. A regenerated cellulose membrane of MWCO 12–14 kDa (Visking dialysis tubing MWCO 12–14 kDa, Medicell Membranes Ltd., London, UK) with an effective surface area of 1.77 cm^2^ was mounted between the donor and acceptor compartments. The regenerated cellulose membrane serves as the standard, simpler, and cheaper barrier often utilized for the transport study and for the sake of comparison between different drug formulations containing a drug compound and excipients. The drug diffusion rate through this membrane is highly controlled by the drug concentration gradient between both sides of the barrier [38,39,40]. The experimental conditions were identical to those used in the dissolution study. The amount of SD complex added to the donor compartment in permeation tests was the same as for the respective dissolution experiment. The concentration of OLZ permeated through the membrane was quantified spectrophotometrically. The permeation experiments meet the sink conditions when the maximum solution concentration in the receiver cell was less than one-third of the crystalline solubility [41].

The cumulative amount of permeated OLZ per unit area (*Q*/*A*) was plotted as a function of time to generate the permeation profiles. Because the donor solution concentration changed over time, the permeability coefficient could not be accurately determined. Therefore, the flux (*J*) was calculated using the following equation:

(4) $$J=\frac{dQ}{A\times dt}$$

### 2.6. Physical Stability Studies

Selected samples were stored under the following conditions to assess their stability: ambient laboratory conditions, 40 °C/ambient humidity, and 75% RH/ambient temperature. In all cases, the samples were protected from light. Stability was monitored by PXRD at different time intervals.

### 2.7. Statistical Analysis

Data are presented as mean ± SD. Differences were analyzed by one-way ANOVA, with a *p*-value ≤ 0.05 considered statistically significant.

## 3. Results and Discussion

### 3.1. Preparation and Characterization of Olanzapine Solid Dispersions

The following solid samples were prepared from raw OLZ for dissolution and permeation studies: ground OLZ (OLZ-gr); OLZ two-component systems (physical mixtures and co-ground SDs in a 1:1 ratio): OLZ/SBE-β-CD, OLZ/GABA, OLZ/6ACA, and OLZ/ChB; and three-component systems OLZ/SBE-β-CD/GABA in 1:0.25:0.75 and 1:0.75:0.25 ratios. The prepared samples were characterized by DSC, PXRD, Raman, and FTIR spectroscopy, and SEM. As emphasized in our previous study [8], various crystalline forms are characteristic of OLZ [15], making the correct identification of the OLZ solid-state forms a crucial step in the formulation process. The raw sample of OLZ was characterized by DSC and PXRD. In our previous study [8], OLZ was shown to be a mixture of Form I and Form II. This was evidenced by distinct PXRD peaks for Form I at 8.68°, 12.6°, 14.88°, 17.1°, 20.24°, 21.54°, and 21.78°; for Form II at 9.06°, 12.98°, 18.62°, 18.98°, 19.9°, 21.1°, and 23.96°; and a peak common to both at 10.47°. In the present work, we compared the characteristics of the prepared SDs with those of the raw OLZ sample.

#### 3.1.1. Characterization of OLZ Solid Preparations

As described above, neat milling was used to prepare the OLZ SDs with the selected excipients. First, the ground OLZ sample was compared with the raw OLZ. For better visualization, the diffractograms of raw OLZ and ground OLZ (OLZ-gr) are presented together in Figure S1 with the most informative regions highlighted. Closer examination revealed that milling raw OLZ transformed the material from a mixture of Forms I and II to a mixture containing Forms I and II with traces of Form III (see Supplementary Materials, Section S2). Interestingly, Bhardwaj [42] reported that the pattern of OLZ Form II (with traces of Form III) was obtained from Form I upon neat grinding.

The PXRD results for the individual excipients, two-component solid dispersions (SD), and corresponding physical mixtures (pm) are shown in Figure S2: (a) with SBE-β-CD, (b) with GABA, (c) with 6ACA, and (d) with ChB. Detailed description of the X-ray diffraction samples is provided in Supplementary Materials, Section S3. The findings provided clear evidence of the significant transformation of OLZ towards a poorly crystallized phase in the OLZ/SBE-β-CD (1:1)-SD (Figure S2a). By inspection of Figure S2b–d, it appears that the crystallinity of OLZ was not reduced upon the formation of physical mixtures with GABA, 6ACA, and ChB. Analysis of the OLZ/GABA (1:1) (Figure S2b), OLZ/6ACA (1:1) (Figure S2c), and OLZ/ChB (1:1) (Figure S2d) indicated that the physical mixture is a simple superposition of the individual OLZ and the excipient. The corresponding solid dispersions exhibited alterations, such as the disappearance or diminished intensity of several characteristic peaks, primarily as an effect of milling rather than the presence of the second component. The state of OLZ and the solid dispersions after dissolution in buffer pH 7.4 were analyzed by PXRD. The results are shown in Figure S2e and described in Section S3. It has been shown that dissolution in the buffer promotes polymorphic transformations of OLZ.

Infrared spectroscopy was used to study the possible microstructural changes upon the formation of the two-component systems. The respective FTIR spectra are given in Figure S3. The main characteristic bands of OLZ and the second component are indicated with black and red lines, respectively, to track potential transformations. The experimental FTIR spectra for the individual excipients, two-component solid dispersions (SD), and corresponding physical mixtures (pm) were comprehensively described (see Supplementary Materials, Section S4). Inspection of Figure S3a revealed that all the characteristic peaks of SBE-β-CD were present in both the pm and SD. The greater intensity of these bands in the SD sample was presumably a consequence of the milling process. All characteristic bands of OLZ were present in the spectra of the physical mixture and SD with GABA and ACA. The principal absorption peaks of ChB, physical mixture, and SD with OLZ are shown in Figure S3d. Only insignificant differences between the spectra of OLZ pm and SD with ChB were observed. FTIR analysis revealed no evidence of interactions between the components in any of the binary solid systems studied.

Raman spectroscopy provides a unique chemical fingerprint for identifying specific compounds. Figure S4 presents the Raman spectra of the prepared two-component solid dispersions and their individual components in the range of 600–2400 cm^−1^. A detailed description of the results for individual excipients and two-component solid dispersions (SD) can be found in Supplementary Materials, Section S5. Analysis of the results suggested the shift in the OLZ band from 1580 cm^−1^ to 1591 cm^−1^ in the OLZ/SBE-β-CD (1:1)-SD serves as an indicator of molecular interaction or structural changes within the system. In contrast, no such interactions were detected with GABA, 6ACA, or ChB. This finding partially contradicts the corresponding FTIR data.

**Figure 2:**
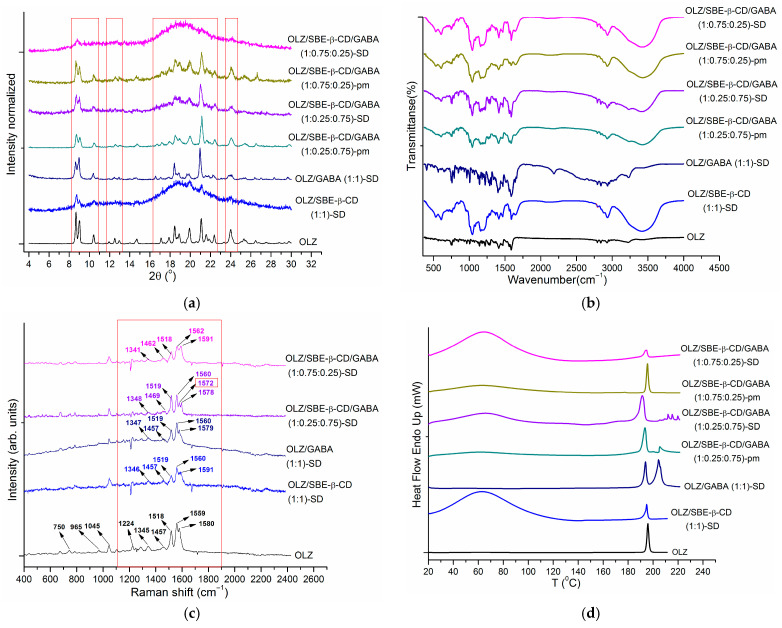
Comparative characterization of the three-component samples: (**a**) PXRD, (**b**) FTIR, (**c**) Raman, and (**d**) DSC.

Differential Scanning Calorimetry was used to investigate the thermal behavior of the samples. As shown in our previous study [8], the raw OLZ sample exhibited a sharp melting peak at 196.4 ± 0.2 °C (T_onset_ = 194.5 ± 0.2 °C), corresponding to Form I. A second endothermic peak in the range of 160–190 °C, partially overlapped by an exothermal peak superposed to it, was consistent with the presence of Form II, as reported in the literature [43]. Both forms were shown to be stable at room temperature for extended periods. The DSC curves of the raw excipients, two- and three-component physical mixtures, and solid dispersions are given in Figure S5a,b and Figure 2d, respectively. The corresponding thermophysical parameters of OLZ are summarized in Table 1.

Analysis of the data revealed the following modifications. The thermophysical parameters of the ground OLZ sample were similar to those of the raw (unground) material. The DSC curves of the two-component physical mixture and SD with SBE-β-CD showed an inflection, corresponding to the desolvation of the cyclodextrin. Only a slight shift in the T_onset_ and T_peak_ of OLZ to lower temperatures was observed. However, a considerable reduction in ∆H_m_—by factors of up to 5.8 and 19.7 for the pm and SD, respectively—was found, indicating a decrease in crystallinity or a phase transition from the crystalline to a partially amorphous form. In the two-component samples with the other excipients, the decrease in melting point was more pronounced than with SBE-β-CD. The enthalpy of fusion ∆H_m_ of OLZ was reduced in the physical mixtures SDs by factors of 1.5 and 2.1 with GABA, 1.8 and 2.4 with 6ACA, and 2.4 and 2.3 with ChB, respectively. The observed thermal behavior, characterized by slight shifts in the melting points of OLZ and the excipients, along with depressed melting enthalpy values in the physical mixtures and even more prominently in the SDs, can be attributed to the reduced crystallinity rather than to the noticeable interactions between OLZ and the excipients. This conclusion is consistent with the PXRD, FTIR, and Raman spectroscopy results.

Next, three-component solid systems (OLZ/SBE-β-CD/GABA) with OLZ/SBE-β-CD/GABA ratios of 1:0.25:0.75 and 1:0.75:0.25 were investigated. The PXRD, FTIR, Raman, and DSC patterns (Figure 2a–d) are presented together with the two-component SDs for comparison. Careful inspection of Figure 2a reveals that the formation of the three-component solid dispersions (SDs) resulted in a further loss of OLZ crystallinity, similar to the trend observed in the two-component systems. Interestingly, the greatest degree of amorphization was observed for the OLZ/SBE-β-CD/GABA (1:0.75:0.25) solid dispersion (SD). Only two OLZ peaks at 2θ = 8.68° and 23.96° remained visible. In contrast, the OLZ/SBE-β-CD (1:1)-SD pattern showed broader, less distinct peaks at 2θ = 8.68°, 9.06°, 10.46°/10.48°, 20.24°, 21.78°, and 23.96°. Moreover, characteristic GABA peaks were detected in all PXRD patterns except for that of the OLZ/SBE-β-CD/GABA (1:0.75:0.25) solid dispersion.

Figure 2b compares the FTIR spectra of the three-component solid samples. The OLZ bands at 3239 cm^−1^ and 2929 cm^−1^ are well-defined in the OLZ/GABA (1:1) pm and SD. However, they become faint in the OLZ/SBE-β-CD/GABA (1:0.25:0.75) systems (both pm and SD) and are completely overlapped by the SBE-β-CD peak in the OLZ/SBE-β-CD/GABA (1:0.75:0.25)-SD. The OLZ bands at 1587 cm^−1^ and 1287 cm^−1^ were present in all studied two- and three-component patterns containing GABA, where they appeared coupled with adjacent acid bands. Finally, the band at 1421 cm^−1^ was observed in all systems studied. In conclusion, the data showed no evidence of strong interactions between OLZ and the excipients in the three-component systems. Alternatively, FTIR spectroscopy may not be sensitive enough to detect such interactions. In contrast, modifications of the characteristic bands were identified in the Raman spectra of the three-component SDs (Figure 2c). A shift in the OLZ band at 1580 cm^−1^ was observed in OLZ/SBE-β-CD (1:1)-SD. A similar shift was identified in the OLZ/SBE-β-CD/GABA (1:0.75:0.25)-SD. In contrast, a new band at 1572 cm^−1^ was observed in the OLZ/SBE-β-CD/GABA (1:0.25:0.75)-SD. This finding confirmed that the structural modifications in the three-component SD arose not only from OLZ-CD interactions but were also influenced by GABA. Thus, Raman spectroscopy proved more effective than IR spectroscopy for detecting interactions in the three-component OLZ/SBE-β-CD/GABA (1:0.25:0.75) solid dispersion.

The DSC results for the three-component OLZ/SBE-β-CD/GABA systems (Figure 2d) demonstrate the influence of the SBE-β-CD:GABA ratio on their thermal behavior. The characteristic OLZ peak was present in all tested samples. The T_peak_ values for the OLZ/SBE-β-CD/GABA (1:0.75:0.25) systems were identical to those of the OLZ/SBE-β-CD (1:1) physical mixtures and solid dispersions (SDs) (Table 1). In contrast, the T_peak_ values for the OLZ/SBE-β-CD/GABA (1:0.25:0.75) pm and SD were shifted to lower temperatures by 2.1 °C and 3.4 °C, respectively. As expected, the ∆H_m_ values for the OLZ/SBE-β-CD/GABA (1:0.25:0.75) systems (both pm and SD) were intermediate between those of the binary OLZ/SBE-β-CD and OLZ/GABA systems. In contrast, the values for the OLZ/SBE-β-CD/GABA (1:0.75:0.25) systems were similar to those of the OLZ/SBE-β-CD system. Notably, ∆H_m_ of OLZ in the OLZ/SBE-β-CD/GABA (1:0.75:0.25) pm was 1.5 kJ·mol^−1^ higher than that in the corresponding OLZ/SBE-β-CD system, whereas in the SD it was 0.8 kJ·mol^−1^ lower. These findings indicated that both the grinding procedure and the presence of GABA promote the transformation of OLZ into a poorly crystalline phase. As is clearly demonstrated in Figure 2d, the melting peak of GABA was visible in the OLZ/SBE-β-CD/GABA (1:0.25:0.75)-pm but was absent in the thermograms of the OLZ/SBE-β-CD/GABA (1:0.25:0.75)-SD, OLZ/SBE-β-CD/GABA (1:0.75:0.25)-pm, and OLZ/SBE-β-CD/GABA (1:0.75:0.25)-SD, in full agreement with the PXRD findings.

#### 3.1.2. Surface Morphology Characterization by SEM

**Figure 3:**
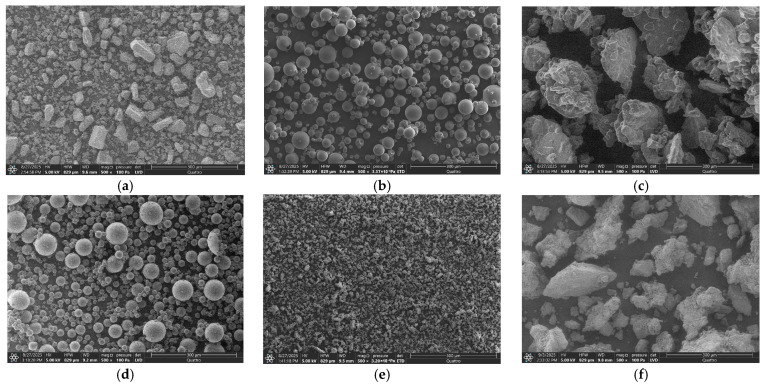

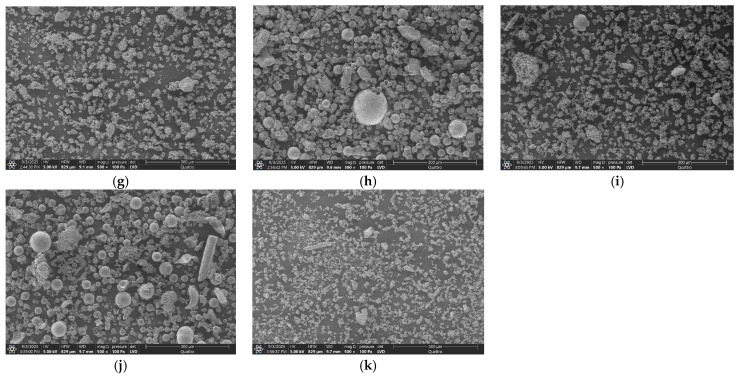
SEM micrographs of raw OLZ (**a**), SBE-β-CD (**b**), GABA (**c**), OLZ/SBE-β-CD (1:1)-pm (**d**), OLZ/SBE-β-CD (1:1)-SD (**e**), OLZ/GABA (1:1)-pm (**f**), OLZ/GABA (1:1)-SD (**g**), OLZ/SBE-β-CD/GABA (1:0.25:0.75)-pm (**h**), OLZ/SBE-β-CD/GABA (1:0.25:0.75)-SD (**i**), OLZ/SBE-β-CD/GABA (1:0.75:0.25)-pm (**j**), and OLZ/SBE-β-CD/GABA (1:0.75:0.25)-SD (**k**). Magnification of ×500 was applied.

This study used Scanning Electron Microscopy (SEM) to characterize morphological changes during the formation of physical mixtures and solid dispersions [44]. SEM imaging was performed at various magnifications, ranging from 500× to 100,000×. For comparison, representative micrographs are shown at 500× (Figure 3) and 1000× (Figure S6). The higher magnification (1000×) was suitable for assessing particle size.

In line with the study of Patil et al. [45], pure OLZ consisted of irregularly shaped crystallites with a lamellar structure and a broad size distribution, ranging from an average of 30 μm up to a maximum of 50 μm (Figure 3a and Figure S6a). SEM analysis revealed that SBE-β-CD consisted of smooth, spherical particles with sizes averaging 30 μm and reaching up to 50 μm (Figure 3b and Figure S6b), which is consistent with the literature [46]. In contrast, GABA (Figure 3c and Figure S6c) appeared as irregular, near-polyhedral blocks with an average size of approximately 100 μm and a maximum of 200 μm [47]. The SEM image of the OLZ/SBE-β-CD (1:1)-pm (Figure 3d and Figure S6d) shows spherical SBE-β-CD particles (of the same size as the raw material) with OLZ crystallites partially attached to their surface. In contrast, the corresponding solid dispersion (Figure 3e and Figure S6e) exhibits an irregular, amorphous morphology where the original shapes of OLZ and SBE-β-CD are no longer discernible, indicating a close association and loss of crystallinity. Analysis of the OLZ/GABA (1:1) images (Figure 3f,g and Figure S6f,g) revealed that the primary difference between the pm and the SD lies in particle size. While both components retained their original morphological shapes, their sizes were reduced by mechanical milling from 30 to 50 μm (OLZ) and 100–200 μm (GABA) to 100–150 μm in the physical mixture and further down to 20–30 μm in the solid dispersion. This particle size reduction was consistent with the PXRD and DSC findings. The SEM micrographs of the three-component OLZ/SBE-β-CD/GABA systems (1:0.25:0.75 and 1:0.75:0.25) showed similar trends. Their physical mixtures appeared as simple blends of the initial components. Upon closer examination, the OLZ/SBE-β-CD/GABA (1:0.25:0.75) physical mixture (Figure 3h and Figure S6h) showed a larger quantity of GABA particles and a smaller quantity of SBE-β-CD particles, which is consistent with the higher GABA ratio in this formulation. The opposite trend was observed in the (1:0.75:0.25) mixture, as expected from its composition. The corresponding SDs (Figure 3i,k and Figure S6i,k) exhibited irregular, amorphous morphologies in which the original shapes of the OLZ and SBE-β-CD components were only faintly discernible. The morphology of OLZ solid dispersions with SBE-β-CD and GABA obtained in our work by the grinding method is fundamentally different from that of olanzapine solid dispersions with polyvinylpyrrolidone K-30 and monoammonium glycyrrhizinate pentahydrate prepared by the spray drying technique, for which a dent-like morphology with the addition of large-size agglomerated particles was observed [45]. For the solid dispersions presented in our work, the main distinguishing feature is the degree of amorphization, which varied depending on the SBE-β-CD content. Notably, in the solid dispersion of OLZ with gelatin, glycine, and sorbitol prepared by dispersing the drug in an aqueous solution of carrier materials and subsequent lyophilization, the micrograph shows a matrix in which no olanzapine crystals could be observed [48].

As expected, the loss of crystallinity was more pronounced in the sample with the higher SBE-β-CD content. A comparison of all SEM micrographs allowed the prepared SDs to be ranked by crystallinity in the following order: OLZ/GABA (1:1)-SD > OLZ/SBE-β-CD/GABA (1:0.25:0.75)-SD > OLZ/SBE-β-CD (1:1)-SD ≥ OLZ/SBE-β-CD/GABA (1:0.75:0.25)-SD. This trend was consistent with the PXRD and DSC results.

### 3.2. In Vitro Dissolution/Release and Diffusion Through the Membrane of OLZ SDs

**Figure 4:**
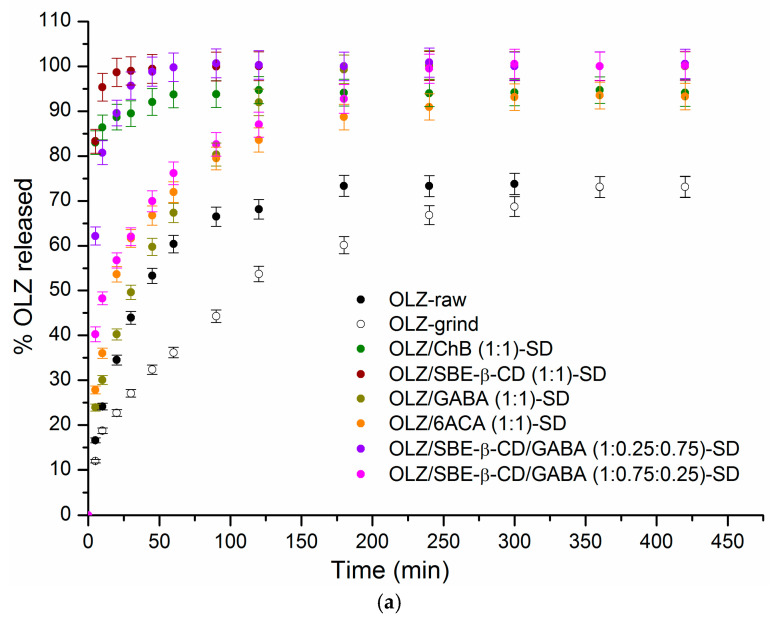

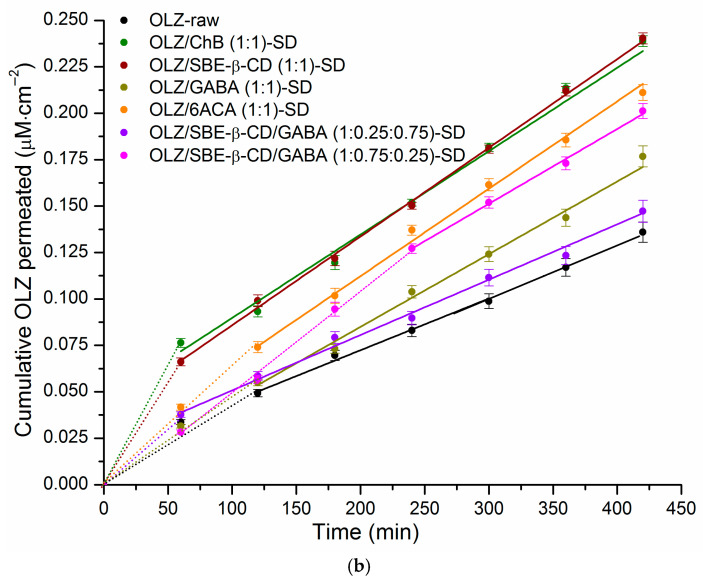
Dissolution (**a**) and permeation (**b**) profiles of OLZ and the prepared solid dispersions at 37 °C.

Controlled drug release from solid dosage forms has garnered sustained research interest. It is well established that crystallinity generally retards dissolution, while the effect of excipients on dissolution and permeation varies with their nature, concentration, and specific drug-excipient interactions [49]. Multicomponent dispersions represent a promising approach to modulating drug release profiles. We investigated the effects of OLZ solid form and excipient composition on its dissolution and membrane permeation. Based on previous findings that OLZ solubility is ~28-fold greater at pH 2.0 than at pH 7.4 [8], we selected pH 7.4 for the dissolution and permeation experiments. The results are presented in Figure 4 as kinetic profiles showing the percentage of OLZ released (Figure 4a) and the cumulative amount of OLZ permeated through a regenerated cellulose membrane (Figure 4b). The dissolution profiles show distinct differences between pure OLZ and the SDs, whose compositions are indicated on the plots. To quantitatively compare the dissolution profiles, several parameters were determined (Table 2): the amount of OLZ released at 5 and 30 min (*Q*_5_/*Q*_30_), the dissolution performance parameter (DPP), the time required to release 85% of OLZ from SDs (*t*_85%_), and the similarity factor (*f*_2_).

Analysis of the drug release data from the solid systems revealed the following findings. The lowest amounts of OLZ dissolved within 5 and 30 min were observed for the raw and ground OLZ solids: 16.6%/43.9% and 12.0%/27.1%, respectively. Moreover, the cumulative release after 420 min (the end of the experiment) was around 73% for raw and ground OLZ, with corresponding dissolution performance values of 67.1% and 57.5%, respectively. This indicates that although grinding reduced the initial dissolution rate of OLZ, it ultimately did not affect the total amount of compound dissolved over the experimental period. The solid dispersions showed a significant improvement in all dissolution parameters compared to the pure drug (Table 2). Based on the t_85%_ values, the following systems can be classified as immediate-release dosage forms, characterized by ≥85% drug dissolution within 30 min: OLZ/SBE-β-CD (1:1) (*t*_85%_ = 5 min), OLZ/ChB (1:1) (*t*_85%_ = 10 min), and OLZ/SBE-β-CD/GABA (1:0.25:0.75) (*t*_85%_ = 20 min) [50]. A similar result was demonstrated for the olanzapine solid dispersion with gelatin, glycine, and sorbitol prepared by the lyophilisation method—95.37% in 5 min [48], in which no crystalline particles of pure olanzapine were observed in the SEM image, and for the olanzapine dispersion with mannitol (1:10), for which *t*_85%_ = 30 min [51].

The rapid release from these formulations is likely due to improved surface wettability and a shorter diffusion path length. For these solid dispersions, the dissolution performance parameter exceeded 90%, with the following values in descending order: OLZ/SBE-β-CD (1:1) at 99.0%, OLZ/SBE-β-CD/GABA (1:0.25:0.75) at 98.4%, and OLZ/ChB (1:1) at 93.0%. Complete (100%) OLZ release was achieved within 60 min for the OLZ/SBE-β-CD (1:1) and OLZ/SBE-β-CD/GABA (1:0.25:0.75) SDs. In contrast, the OLZ/GABA (1:1) and OLZ/SBE-β-CD/GABA (1:0.75:0.25) SDs required 240 min and 300 min, respectively, to reach the same release level. For comparison, in the study by Jeet et al. [52], for a solid dispersion (fusion method) containing 200 mg each of PVP K30 and PEG 6000, the maximum amount of olanzapine released within 60 min was 56.71%. In the ternary SDs in our study, the amounts of SBE-β-CD and GABA were 0.65 mg and 0.139 mg (OLZ/SBE-β-CD/GABA (1:0.25:0.75)) and 1.91 mg and 0.045 mg (OLZ/SBE-β-CD/GABA (1:0.75:0.25)), respectively.

A comparison of the release profiles from the three-component SDs revealed that the release rate and kinetics were influenced not only by the choice of excipients but also by their ratio. Despite these differences in release rate, complete (100%) OLZ dissolution was ultimately achieved in both three-component SDs. Specifically, a higher GABA content promoted immediate release, whereas a greater proportion of SBE-β-CD retarded dissolution. Interestingly, the corresponding two-component solid dispersions also achieved complete (100%) OLZ release, albeit at different rates: OLZ/SBE-β-CD (1:1) required only 5 min for 85% release and 60 min for complete release, whereas OLZ/GABA (1:1) required 120 min and 240 min, respectively. The Raman spectrum of the OLZ/SBE-β-CD/GABA (1:0.25:0.75) solid dispersion showed a new band at 1572 cm^−1^, which was attributed to specific molecular interactions between the three components that occurred uniquely at this particular ratio. These interactions likely account for the distinct dissolution behavior of the OLZ/SBE-β-CD/GABA (1:0.25:0.75)-SD. The PXRD pattern of the OLZ/SBE-β-CD/GABA (1:0.75:0.25) solid dispersion showed only two OLZ peaks (at 2θ = 8.68° and 23.96°), an absence of characteristic GABA peaks, and a maximal loss of crystallinity. This finding was corroborated by the DSC data.

Table 2 presents the *f*_2_ similarity factors for the dissolution curves in Figure 4, with OLZ-raw as the reference. All experimental points (13 points) were used for the *f*_2_ similarity analysis. The RSD values for the 20 min (initial period) and 360 min (final period) time points were 3.21% and 3.19%, respectively. As stated, *f*_2_ values greater than 50 indicate similarity between the dissolution profiles [53]. The data presented in Table 2 indicate that the least difference was found between OLZ-raw and OLZ-grind. In contrast, none of the tested solid dispersions exhibited a profile similar to the reference. The similarity of the SDs to OLZ-raw decreased in the following order: OLZ/6ACA (1:1) [*f*_2_ = 39.5] > OLZ/GABA (1:1) [36.4] > OLZ/SBE-β-CD/GABA (1:0.75:0.25) [32.8] > OLZ/ChB (1:1) [20.6] ≥ OLZ/SBE-β-CD/GABA (1:0.25:0.75) [20.1] > OLZ/SBE-β-CD (1:1) [17.5]. To evaluate the similarity between the two-component and corresponding three-component SDs, the similarity factor was calculated using OLZ/SBE-β-CD (1:1) as the reference (Table 2). First, the two-component solid dispersions OLZ/SBE-β-CD (1:1) and OLZ/GABA (1:1) showed poor similarity (*f*_2_ = 22.3). The similarity to OLZ/SBE-β-CD (1:1) was influenced by the SD composition. The OLZ/SBE-β-CD/GABA (1:0.25:0.75)-SD showed good similarity (*f*_2_ = 55.7), while the formulation with a higher SBE-β-CD ratio (1:0.75:0.25) showed markedly lower similarity (*f*_2_ = 28.8). In summary, the release behavior of OLZ from the three-component SDs appeared to depend more on the complex interactions between the components than on their quantitative ratio. Markedly, the characterization of the SDs by PXRD, Raman spectroscopy, and DSC revealed the critical role of mutual interactions between components.

**Figure 5:**
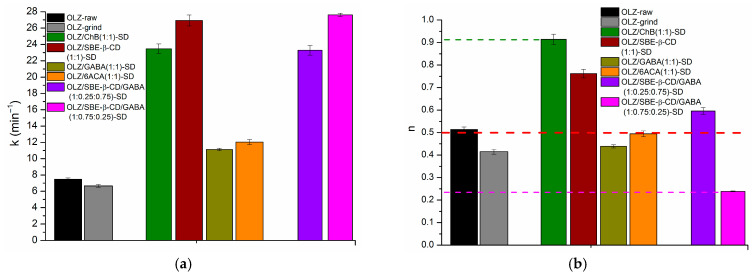
(**a**) Release constant (*k*) and (**b**) transport exponent (*n*), derived from the non-linear fitting of drug release data to Equation (3). Data are presented as mean ± SD (*n* ≥ 3).

The drug release from solid dispersions containing polymeric excipients is a complex process involving water diffusion, polymer swelling/erosion, excipient dissolution, and drug diffusion into the bulk solution. This process is often described by the Korsmeyer–Peppas model [54]. In this study, the simple power-law equation of the Korsmeyer–Peppas model was used to investigate the kinetics and mechanism of OLZ release from the SDs. Mathematical computations performed in Microsoft Excel demonstrated high correlation coefficients ranging from R^2^ = 0.9938 for the GABA(1:0.25:0.75)-SD to R^2^ = 0.9999 for the OLZ/SBE-β-CD GABA(1:0.25:0.75)-SD. Figure 5 illustrates the release constant (*k*), reflecting structural and geometric characteristics, and the diffusional exponent (*n*), describing the release mechanism, for all solid dispersions.

Figure 5a shows that the release rate constant (*k*) was highest for OLZ/SBE-β-CD/GABA (1:0.75:0.25)-SD (27.6 min^−n^) and OLZ/SBE-β-CD (1:1) SD (26.9 min^−n^), and lowest for OLZ-raw (7.5 min^−n^) and OLZ-grind (6.7 min^−n^), which is consistent with the other release parameters. In turn, Jeet et al. [52] obtained a maximum rate constant of 12.9 min^−1^ for a solid dispersion composed of β-cyclodextrin (100 mg) + PEG 6000 (400 mg), which proved to be 2.2 times lower than that for the OLZ/SBE-β-CD/GABA (1:0.75:0.25)-SD obtained in our work. Diffusion exponent values of n ≈ 0.5 for OLZ-raw, OLZ/6ACA (1:1)-SD, and n < 0.5 for OLZ-grind, OLZ/GABA (1:1)-SD, and OLZ/SBE-β-CD/GABA (1:0.75:0.25)-SD confirmed the dominance of the Fickian diffusion mechanism. In the literature, the values of exponent *n* < 0.5 were determined for solid dispersions of olanzapine:mannitol (up to 1:10, *n* = 0.342) [51]. This mechanism is characterized by a diminishing drug concentration gradient over time, where this gradient serves as the driving force for a controlled release process [55,56]. For OLZ/SBE-β-CD/GABA (1:0.25:0.75)-SD (*n* = 0.60), OLZ/SBE-β-CD (1:1)-SD (*n* = 0.76), and OLZ/ChB (1:1)-SD (*n* = 0.91), the release mechanism was governed by non-Fickian (anomalous) transport, which is controlled by both diffusion and swelling/erosion [36]. Similar values of *n* were obtained in the literature [52] for olanzapine solid dispersions with various ratios of PVP K30, PEG 6000, and β-cyclodextrin, prepared by solvent evaporation and the fusion method. Notably, the *n*-value for OLZ/ChB (1:1)-SD was closest to 1, suggesting its release kinetics partially follow a zero-order model, characterized by a constant release rate independent of concentration.

Figure 4b shows the permeation profiles, and Table 3 lists the corresponding quantitative parameters.

Careful inspection of Figure 4b reveals that the cumulative amount of permeated OLZ increased more rapidly during the initial time period. The flux changed at 60 min in the systems with the fastest initial dissolution: OLZ/ChB (1:1)-SD, OLZ/SBE-β-CD (1:1)-SD, and OLZ/SBE-β-CD/GABA (1:0.25:0.75)-SD. For the remaining solid samples, this transition was observed at 120 min for OLZ-raw, OLZ/GABA (1:1)-SD, and OLZ/6ACA (1:1)-SD, and at 240 min for OLZ/SBE-β-CD/GABA (1:0.75:0.25)-SD. To compare the overall dissolution and permeation trends of OLZ, the cumulative amount permeated at 420 min ($Q_{420}^{perm}$) and the ratio of dissolved to permeated amount at 420 min (*Q*_*ratio*_) were determined and are listed in Table 3. Based on the $Q_{420}^{perm}$ values, the studied SDs were ranked as follows: OLZ/SBE-β-CD (1:1) ≈ OLZ/ChB (1:1) > OLZ/6ACA (1:1) ≥ OLZ/SBE-β-CD/GABA (1:0.75:0.25) > OLZ/GABA (1:1) > OLZ/SBE-β-CD/GABA (1:0.25:0.75) > OLZ-raw. The largest difference (6.92) between the dissolved and permeated amounts of OLZ was observed for the OLZ/SBE-β-CD/GABA (1:0.25:0.75)-SD, whereas the OLZ/SBE-β-CD/GABA (1:0.75:0.25)-SD had a *Q*_*ratio*_ of 5.05. Considering the *Q*_*ratio*_ values for the corresponding two-component SDs (4.21 for OLZ/SBE-β-CD (1:1)-SD and 5.73 for OLZ/GABA (1:1)-SD), it appears that a higher proportion of GABA reduces OLZ permeation, whereas a higher proportion of SBE-β-CD enhances it. In the literature, there is only one article that discusses the membrane permeability of olanzapine through artificial cellulose acetate and cellulose nitrate membranes [51]. As established by the authors, for the OLZ:mannitol SD (1:10), the apparent permeation coefficient increased by 8 and 9 times, respectively, when using cellulose acetate and cellulose nitrate membranes. Unfortunately, complete permeability profiles were not provided in that study, rendering a correct comparison of the membrane diffusion processes with our data impossible.

### 3.3. Stability Tests

The PXRD spectra of the selected samples after storage under different conditions are provided in Figure S7. As shown in Figure S7a, the two-component solid dispersions (OLZ/SBE-β-CD and OLZ/GABA) and the three-component (OLZ/SBE-β-CD/GABA (1:0.25:0.75)) remained physically stable, showing no detectable changes in their diffraction patterns after 3 months of storage under ambient conditions. The OLZ-raw material also remained unchanged under ambient conditions. The diffractogram of pure OLZ stored at 40 °C and ambient relative humidity (Figure S7b) showed no physical transformations for up to 16 days. In contrast, the sample stored at 75% relative humidity (Figure S7c) exhibited only minor changes after 72 days. The stability study of the freshly prepared OLZ/SBE-β-CD (1:1) SD revealed that high humidity introduced moisture into the system, rendering the samples unsuitable for further testing. In contrast, storage at 40 °C (Figure S7d) for up to 72 days did not affect the chemical stability of the dispersion. For OLZ/GABA (1:1)-SD (Figure S7e), elevated temperature gradually increased the crystallinity, as evidenced by the growing intensity of the OLZ peaks at 2θ = 8.68°, 9.06°, 12.6°, and 19.9°. This effect was also observed at 75% RH (Figure S7f), but only after 72 days. The observed increase in crystallinity may be attributed to enhanced molecular mobility at elevated temperatures, facilitating recrystallization. Stability testing confirmed that the OLZ/SBE-β-CD/GABA (1:0.25:0.75) SD (Figure S7g) remained physically stable at 40 °C for 72 days. Although exposure to humidity increased the crystallinity of the OLZ/SBE-β-CD/GABA (1:0.25:0.75) SD, the sample remained suitable for processing up to 16 days. However, after 72 days, the samples absorbed moisture and became unsuitable for further testing.

In summary, the stability study of the selected samples yielded the following conclusions. Ambient conditions were the most favorable for the storage of the obtained solid dispersions. High humidity induced significant changes in the physical state of the OLZ/SBE-β-CD (1:1) and the OLZ/SBE-β-CD/GABA (1:0.25:0.75) solid dispersions. The incorporation of GABA into this system enhanced its stability, despite causing a moderate increase in crystallinity. In contrast, the three-component OLZ/SBE-β-CD/GABA (1:0.25:0.75) SD was more susceptible to the effects of elevated temperature.

## 4. Conclusions

In this study, the release and permeation rate of olanzapine (OLZ) were significantly enhanced using the solid dispersion (SD)-based approach. Two-component (1:1) solid dispersions (OLZ/SBE-β-CD, OLZ/GABA, OLZ/6ACA, OLZ/ChB) and three-component systems (OLZ/SBE-β-CD/GABA at 1:0.25:0.75 and 1:0.75:0.25 ratios) were prepared by mechanical grinding. Analytical characterization confirmed a significant conversion of olanzapine (OLZ) to a poorly crystalline phase within the OLZ/SBE-β-CD solid dispersion (SD). The Raman spectrum of the OLZ/SBE-β-CD/GABA (1:0.25:0.75) solid dispersion showed features suggestive of molecular interactions between the components. Based on Raman and DSC data, the crystallinity reduction in this mixed dispersion is attributed to the synergistic effects of milling and the presence of both SBE-β-CD and GABA. The study highlighted the importance of a multi-faceted analytical approach, which employed various techniques, to more accurately identify potential interactions between the components of solid dispersions.

The OLZ/SBE-β-CD (1:1), OLZ/ChB (1:1), and OLZ/SBE-β-CD/GABA (1:0.25:0.75) systems were classified as immediate-release dosage forms, characterized by the dissolution of not less than 85% of the active pharmaceutical ingredient within 30 min. Notably, the OLZ/SBE-β-CD (1:1), OLZ/GABA (1:1), and both OLZ/SBE-β-CD/GABA solid dispersions ((1:0.25:0.75) and (1:0.75:0.25)) achieved complete release (100%) of olanzapine within 7 h, with a dissolution performance (DPP) of ≥90%. The flux of olanzapine across the membrane was enhanced for all solid dispersions compared to the pure drug. In the ternary dispersions, a higher GABA proportion reduced OLZ permeation, while a higher SBE-β-CD proportion enhanced it; that is fully consistent with the behavior of the corresponding binary complexes.

According to the stability tests, the OLZ/SBE-β-CD (1:1) dispersion was shown to be susceptible to moisture, whereas the three-component OLZ/SBE-β-CD/GABA one was stable against humidity but sensitive to temperature. Therefore, the “normal conditions” represented a compromise that ensured the highest stability for all systems.

The relationships identified between composition, structure, and performance in this study can guide the development of optimized olanzapine solid dosage forms.

## Figures and Tables

**Table 1:** Thermophysical parameters of untreated and processed OLZ.

Sample	T_onset_ (°C)	T_peak_ (°C)	∆H_m_ (kJ∙mol^−1^)
OLZ	194.5 ± 0.2	196.4 ± 0.2	37.4 ± 0.2
OLZ-gr	194.3 ± 0.2	195.7 ± 0.2	36.7 ± 0.2
Two-component systems			
OLZ/SBE-β-CD-pm	194.2 ± 0.2	195.6 ± 0.2	6.5 ± 0.2
OLZ/SBE-β-CD-SD	193.4 ± 0.2	194.8 ± 0.2	1.9 ± 0.2
OLZ/GABA-pm	192.9 ± 0.2	194.5 ± 0.2	24.2 ± 0.2
OLZ/GABA-SD	191.9 ± 0.2	193.9 ± 0.2	17.8 ± 0.2
OLZ/6ACA-pm	190.6 ± 0.2	192.7 ± 0.2	21.3 ± 0.2
OLZ/6ACA-SD	188.4 ± 0.2	190.6 ± 0.2	15.4 ± 0.2
OLZ/ChB-pm	187.4 ± 0.2	191.7 ± 0.2	15.9 ± 0.2
OLZ/ChB-SD	188.0 ± 0.2	191.5 ± 0.2	16.0 ± 0.2
Three-component systems			
OLZ/SBE-β-CD/GABA (1:0.25:0.75)-pm	191.4 ± 0.2	193.5 ± 0.2	16.2 ± 0.2
OLZ/SBE-β-CD/GABA (1:0.25:0.75)-SD	187.6 ± 0.2	191.4 ± 0.2	15.0 ± 0.2
OLZ/SBE-β-CD/GABA (1:0.75:0.25)-pm	193.8 ± 0.2	195.6 ± 0.2	7.5 ± 0.2
OLZ/SBE-β-CD/GABA (1:0.75:0.25)-SD	191.3 ± 0.2	194.9 ± 0.2	1.1 ± 0.2

**Table 2:** Quantitative parameters of dissolution/release of pure OLZ and solid dispersions.

System	*Q*_5_/*Q*_30_ ^1^	DPP ^2^	*t*_85%_ ^3^	*t*_100%_ ^3^	*f*_2_ ^4^	*f*_2_ ^4^
OLZ-raw	16.6/43.9	67.1	-	-	Ref.	-
OLZ-grind	12.0/27.1	57.5	-	-	43.1	-
OLZ/ChB (1:1)	83.0/89.5	93.0	10	-	20.6	-
OLZ/SBE-β-CD (1:1)	82.3/99.0	99.0	5	60	17.5	Ref.
OLZ/GABA (1:1)	23.9/49.6	88.8	120	240	36.4	22.3
OLZ/6ACA (1:1)	27.8/61.6	83.9	180	-	39.5	-
OLZ/SBE-β-CD/GABA (1:0.25:0.75)	62.2/95.7	98.4	20	60	20.1	55.7
OLZ/SBE-β-CD/GABA (1:0.75:0.25)	40.3/62.0	89.6	120	300	32.8	28.8

^1^ *Q*_5_/*Q*_30_ (%)—the amount of OLZ released at 5 and 30 min (%); ^2^ DPP—the dissolution performance parameter calculated by Equation (1); ^3^ t_85%_ and t_100%_ (min)—the time taken to release 85% and 100% of OLZ from SDs; ^4^ *f*_2_—the similarity factor calculated by Equation (2).

**Table 3:** Quantitative parameters of OLZ permeation from the raw sample and solid dispersions.

System	*J*(1) ^1^	*J*(2) ^1^	*t* ^2^	$Q_{420}^{perm}$ ^3^	*Q*_*ratio*_ ^4^
OLZ-raw	9.36 × 10^−6^	4.68 × 10^−6^	120	7.52 × 10^−2^	5.45
OLZ/ChB (1:1)	2.12 × 10^−5^	7.84 × 10^−6^	60	1.32 × 10^−1^	4.39
OLZ/SBE-β-CD (1:1)	1.84 × 10^−5^	8.01 × 10^−6^	60	1.33 × 10^−1^	4.21
OLZ/GABA (1:1)	8.74 × 10^−6^	6.59 × 10^−6^	120	9.78 × 10^−2^	5.73
OLZ/6ACA (1:1)	1.03 × 10^−5^	7.64 × 10^−6^	120	1.17 × 10^−1^	4.46
OLZ/SBE-β-CD/GABA (1:0.25:0.75)	1.05 × 10^−5^	4.87 × 10^−6^	60	8.14 × 10^−2^	6.92
OLZ/SBE-β-CD/GABA (1:0.75:0.25)	8.90 × 10^−6^	6.76 × 10^−6^	240	1.11 × 10^−1^	5.05

^1^ *J*(1) and *J*(2) (µmol∙cm^−2^∙sec^−1^)—the fluxes of OLZ through the membrane for the first and the second time period, respectively; ^2^ *t* (min)—a time point of flux decrease; ^3^ $Q_{420}^{perm}$ (mg)—cumulative OLZ diffused through the membrane at 420 min; ^4^ $Q_{ratio}=Q_{420}^{diss}/Q_{420}^{perm}$ (ratio between the OLZ cumulative amount dissolved and permeated at 420 min).

## Data Availability

The original contributions presented in this study are included in the article and Supplementary Material. Further inquiries can be directed to the corresponding author.

## Supplementary Materials

The following supporting information can be downloaded at https://www.mdpi.com/article/10.3390/pharmaceutics18040411/s1, Section S1: Characterization techniques [57]; Figure S1: Illustration of OLZ raw transformations upon grinding; Section S2: Identification and crystal structure analysis of ground OLZ; Figure S2. Powder X-ray diffraction profiles of individual compounds: raw OLZ (OLZ), ground OLZ (OLZ-gr), SBE-β-CD (CD), γ-aminobutyric acid (GABA), 6-aminocaproic acid (6ACA), choline bitartrate (ChB); physical mixtures: OLZ/CD-pm, OLZ/GABA-pm, OLZ/6ACA-pm, OLZ/ChB-pm; solid dispersions: OLZ/CD-SD, OLZ/GABA-SD, OLZ/6ACA-SD, OLZ/ChB-SD. (a) systems with SBE-β-CD, (b) systems with GABA, (c) systems with 6ACA, (d) systems with ChB, and (e) solid residuals after the dissolution. The most informative regions of diffractograms are marked by rectangles; Section S3: Identification and crystal structure analysis of individual excipients and two-component systems; Figure S3: Infrared spectra of individual compounds: raw OLZ (OLZ), SBE-β-CD (CD), γ-aminobutyric acid (GABA), 6-aminocaproic acid (6ACA), choline bitartrate (ChB); physical mixtures: OLZ/CD-pm, OLZ/GABA-pm, OLZ/6ACA-pm, OLZ/ChB-pm; solid dispersions: OLZ/CD-SD, OLZ/GABA-SD, OLZ/6ACA-SD, OLZ/ChB-SD: (a) systems with SBE-β-CD, (b) systems with GABA, (c) systems with 6ACA, (d) systems with ChB; Section S4: Description of the IR spectra of individual excipients and two-component systems [51,58,59]; Figure S4: Raman spectra of excipients: SBE-β-CD (CD), γ-aminobutyric acid (GABA), 6-aminocaproic acid (6ACA), choline bitartrate (ChB) (a), and solid dispersions: OLZ/SBE-β-CD (1:1)-SD, OLZ/GABA (1:1)-SD, OLZ/6ACA (1:1)-SD, OLZ/ChB (1:1)-SD (b); Section S5: Description of the Raman spectra of individual excipients and two-component systems [42,43,51,60]; Figure S5: DSC patterns of excipients: SBE-β-CD (CD), γ-aminobutyric acid (GABA), 6-aminocaproic acid (6ACA), choline bitartrate (ChB) (a), two-component solid samples: physical mixtures (pm) and solid dispersions (SD) with OLZ (b); Figure S6: SEM micrographs of raw OLZ (a), SBE-β-CD (b), GABA (c), OLZ/SBE-β-CD (1:1)-pm (d), OLZ/SBE-β-CD (1:1)-SD (e), OLZ/GABA (1:1)-pm (f), OLZ/GABA (1:1)-SD (g), OLZ/SBE-β-CD/GABA (1:0.25:0.75)-pm (h), OLZ/SBE-β-CD/GABA (1:0.25:0.75)-SD (i), OLZ/SBE-β-CD/GABA (1:0.75:0.25)-pm (j), OLZ/SBE-β-CD/GABA (1:0.75:0.25)-SD (k). Applied magnification was (×1000). The maximal sizes/average sizes of the particles where applicable are indicated just below the respective images; Figure S7: Stability at different storage conditions: selected SDs at ambient conditions (a), OLZ, T = 40 °C/ambient RH (b), OLZ, RH = 75%/ambient temperature (c), OLZ/SBE-β-CD (1:1), T = 40 °C/ambient RH (d), OLZ/GABA (1:1), T = 40 °C/ambient RH (e), OLZ/GABA (1:1), RH = 75%/ambient temperature (f), OLZ/SBE-β-CD/GABA (1:0.25:0.75)-SD, T = 40 °C/ambient RH (g), OLZ/SBE-β-CD/GABA (1:0.25:0.75)-SD, RH = 75%/ambient temperature (h); Table S1: Sample Table.
