# Supplementary material for: Mechanochemical Activation of Olanzapine in Mixed Solid Dispersions: Impact of Excipients on Release and Permeation Rates
Source: Pharmaceutics. 2026 Mar 27;18(4):411. doi: 10.3390/pharmaceutics18040411 (PMC13119049; doi:10.3390/pharmaceutics18040411)
Supplement: Supplementary file 1 [file pharmaceutics-18-00411-s001.zip › pharmaceutics-4205611-supplementary.pdf]

Supporting Information

Mechanochemical activation of olanzapine in mixed solid dispersions: Impact of excipients on release and permeation rate

Tatyana V. Volkova*, Olga R. Simonova, German L. Perlovich

G.A. Krestov Institute of Solution Chemistry RAS, 153045 Ivanovo, Russian Federation

**Corresponding author: 1 Akademicheskaya str., 153045 Ivanovo, Russian Federation,*

E-mail: vtv@isc-ras.ru

Section S1. Characterization techniques

The PXRD tests were recorded under ambient conditions on a D2 Phaser (Bragg Brentano) diffractometer (Bruker AXS, Karlsruhe, Germany) equipped with a copper X-ray source ($\lambda CuK\alpha 1$=1.5406Å) and a high-resolution position-sensitive LYNXEYE XE T detector. The samples were placed in plate holders and rotated at 15 rpm during data acquisition.

The FTIR spectra of the solid dispersion, physical mixture, and raw OLZ were recorded with the help of a VERTEX 80 v FTIR spectrometer (Billerica, Massachusetts, USA). The samples were pressed into KBr pellets, and scanned over a wavenumber range of 4000–350 cm^{-1}.

Raman spectra were acquired on a Confotec NR500 confocal Raman microscope (Republic of Belarus, 220005, Minsk), using a 532 nm laser. To improve the signal-to-noise ratio, the laser power was attenuated.

Thermal analysis was carried out on a PerkinElmer DSC 4000 system (PerkinElmer DSC 4000, Analytical Instruments, Norwalk, Connecticut, USA) equipped with a refrigerated cooling system. Samples of ~3 mg were weighed using an analytical balance A&D (GR-202) (Japan) with an uncertainty of ±0.05 mg. The following conditions were used: a flowing dry helium atmosphere (20 $cm^3 \cdot min^{-1}$ purity ≥99.996 %, standard aluminum sample pans, and a heating rate of 10 $K \cdot min^{-1}$. The DSC apparatus was calibrated using a two-point method, with indium and zinc standards. The scan-rate-independent melting onset was used for calibration. The melting temperatures of indium and zinc were determined to be 156.6 °C and 419.5 °C, respectively (mean of at least ten measurements). Our results are consistent with the recommended literature data [1].

The surface morphology of the samples was studied using a Thermo Fisher Scientific Quattro S high-performance scanning electron microscope (Brno, Czech Republic). An acceleration voltage of 20–30 kV was applied; micrographs were recorded at several magnifications. The most representative micrographs are presented and discussed.

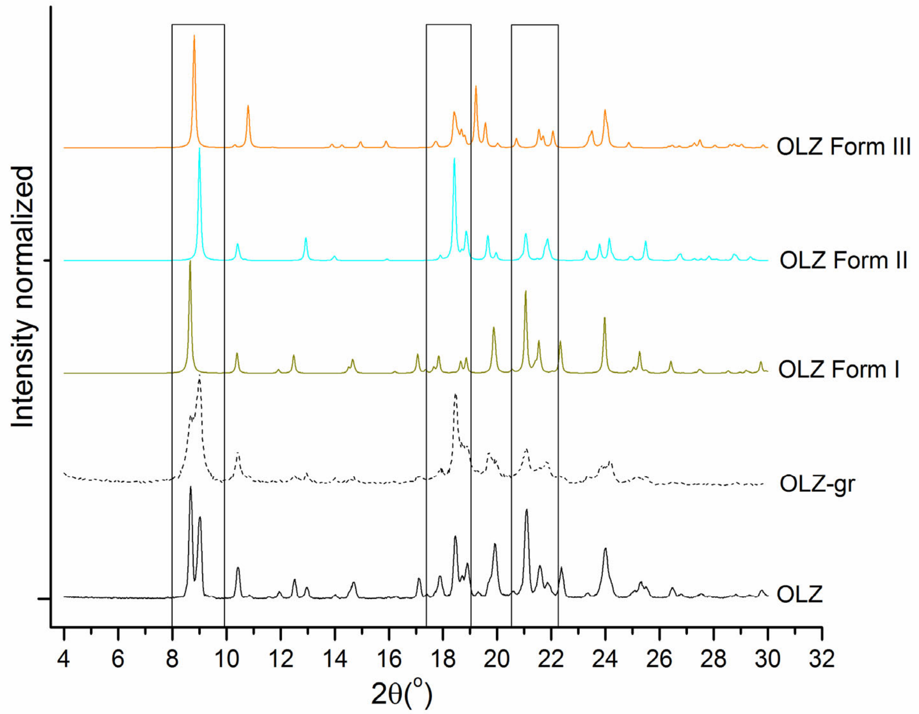

Figure S1. Illustration of OLZ raw transformations upon grinding.

Section S2. Identification and crystal structure analysis of ground OLZ

New peaks at 13.81°, 18.47°, and 19.56° were identified as being characteristic of Form III. The peaks characteristic of Form I (12.47°, 17.11°, 21.78°) and Form II (9.06°, 18.98°, 21.02°) were also detected. Furthermore, the ground OLZ sample also contained peaks at 10.47° (common to Forms I and II) and at 24.0° (common to Forms II and III) (Fig. S1). Additionally, it was shown that the Form I peak at 8.68° was poorly visible, while the intensity of the Form II peak at 9.06° increased. The intensities of the peaks at 12.6° (Form I), 12.98° (Form II), 14.88° (Form I), and 17.1° (Form I) were significantly smoothed.

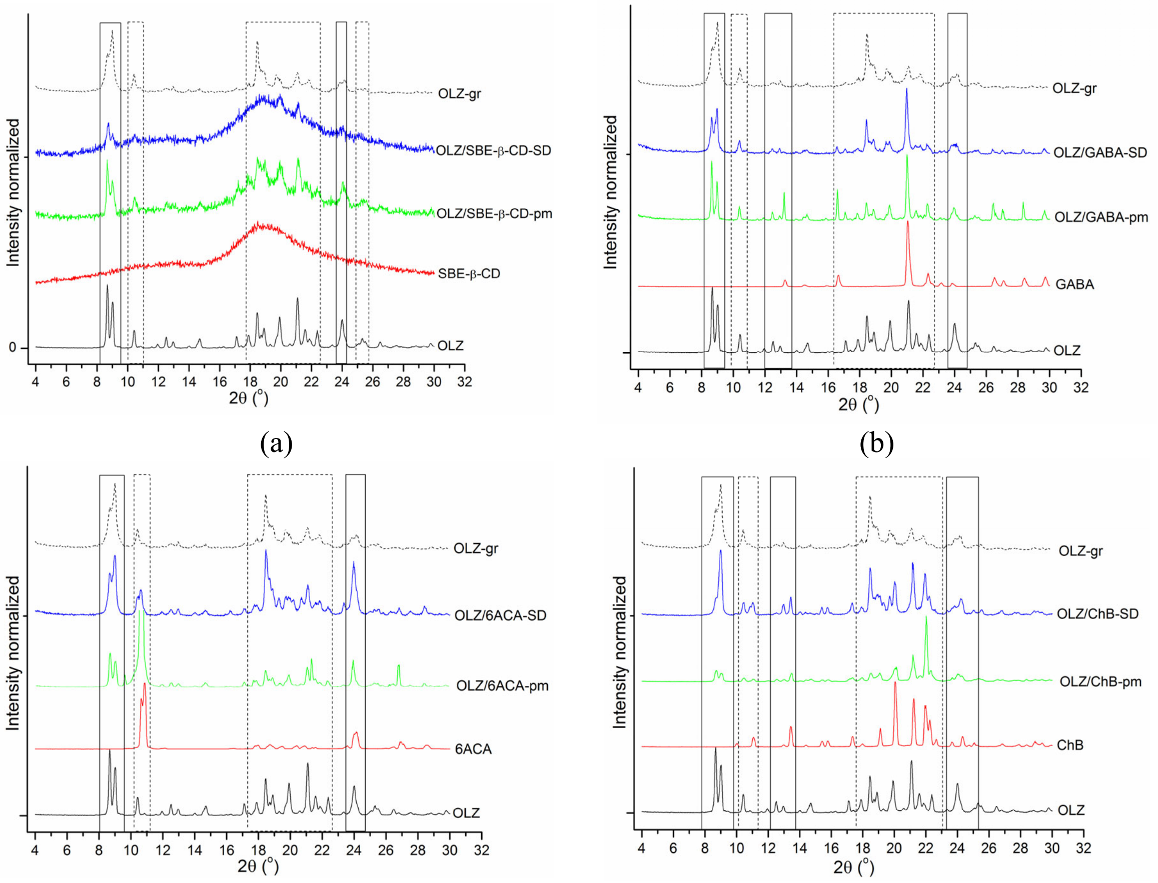

(a) (b)

(c) (d)

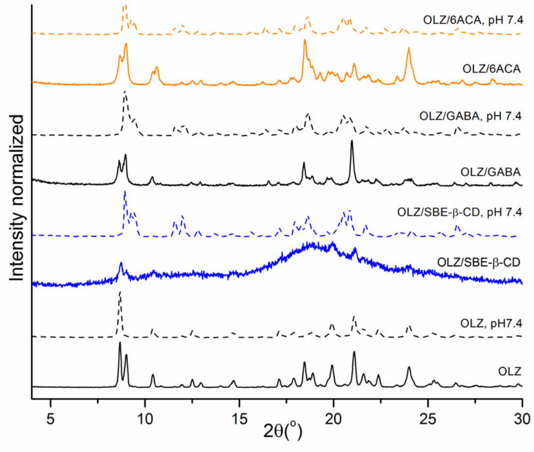

(e)

Figure S2. Powder X-Ray diffraction profiles of individual compounds: raw OLZ (OLZ), ground OLZ (OLZ-gr), SBE-β-CD (CD), γ-aminobutyric acid (GABA), 6-aminocaproic acid (6ACA), choline bitartrate (ChB); physical mixtures: OLZ/CD-pm, OLZ/GABA-pm, OLZ/6ACA-pm, OLZ/ChB-pm; solid dispersions: OLZ/CD-SD, OLZ/GABA-SD, OLZ/6ACA-SD, OLZ/ChB-SD. (a) systems with SBE-β-CD, (b) systems with GABA, (c) systems with 6ACA, (d) systems with ChB, (e) solid residuals after the dissolution. The most informative regions of diffractograms are marked by rectangles.

Section S3. Identification and crystal structure analysis of individual excipients and two-component systems

Fig. S2a illustrates the changes in the OLZ pattern upon simple mixing and milling to obtain the solid dispersion OLZ/SBE-β-CD-SD. A dome-shaped region in the 2θ range from 15° to 25° reaffirmed the amorphous state of SBE-β-CD. The main characteristic peaks of

OLZ Form I and Form II (the most pronounced at 8.68°, 9.06°, 10.46°/10.48°, 17.1°, 18.62°, 19.7°, 20.24°, 21.78°, 23.96°) were visible in the physical mixture with CD. These peaks were accompanied by some amorphization, possibly due to the partial dispersion of crystalline OLZ within the amorphous SBE-β-CD phase. In the SD, however, only the peaks at 8.68°, 9.06°, 10.46°/10.48°, 20.24°, 21.78°, and 23.96° were visible, with marginally reduced intensity.

Analysis of the OLZ/GABA system (Fig. S2b) revealed the following. The PXRD pattern of GABA (Fig. S2b) demonstrated the characteristic peaks at 2θ=13.22°, 14.65°, 16.58°, 20.98°, 22.28°, 23.12°, 23.84°, 26.45°, 27.01°, 28.33°, and 29.69°. The peaks at 20.98°, 22.28° and 29.69° were unchanged in both the pm and SD. The peak at 13.22°, which was visible in the PM, was absent in the SD. Furthermore, the intensities of the peaks at 16.58°, 26.45°, 27.01°, and 28.33° were reduced in the SD. In the physical mixture, the peak at 8.68° (Form I) was more intense than the peak at 9.06° (Form II), similar to the raw OLZ sample. In the SD, however, the relative intensities were reversed. Given that the ground OLZ sample exhibited only a single peak at 2θ=9.06° (Form II), possibly, the behavior of OLZ in the SD appeared to be primarily influenced by the processing method. The peaks of OLZ at 10.48°, 12.6°, 17.1°, 18.62°, 18.98°, 20.98°, and 23.96° were presented in both the pm and SD, appearing broader in the SD.

The PXRD pattern of 6ACA (Fig. S2c) showed the main characteristic peaks at 10.65°, 10.89°, 24.14°, 26.94°, and 28.51°. The intensities of the 6ACA peaks at 10.65°, 26.94°, and 28.51° were reduced in the SD compared to the pm. Changes in the characteristic OLZ peaks after the formation of the PM and SD are summarized below. The peaks at 8.68° (Form I) and 9.06° (Form II) were present in both the pm and SD. However, in the SD, the intensity of the peak at 8.68° decreased, while the intensity at 9.06° increased, a trend similar to that observed in the OLZ/GABA system. The peaks at 10.48 ° and 19.9 ° were not visible in the pm or SD, whereas the peak at 21.54 ° was observed in the pm but broadened in the SD. The OLZ peak at 23.96° was overlapped in the PM by the 6ACA peak at 24.14° but was resolved in the SD.

The diffraction pattern of ChB (Fig. S2d) was characterized by noticeable peaks at 11.09°, 13.45 °, 15.42 °, 15.82 °, 17.38 °, 19.10 °, 20.00 °, 21.25 °, 21.94 °, 22.23 °, 22.69 °, and 24.36 °. As shown in Fig. S2d, most characteristic ChB peaks (at 11.09 °, 13.45 °, 15.42 °, 15.82 °, 17.38 °, 20.00 °, 21.25 °, 21.94 °, and 22.23 °) were visible in both the pm and SD. However, the peak at 19.10 ° was visible in the pm but became broadened in the SD, the one

at 22.69 ° disappeared in both the pm and SD. Additionally, the peak at 24.36 ° merged with the OLZ peak at 23.96 ° to form a broad event ranging from 23.80 ° to 24.55 °.

Since the existence of numerous polymorphic modifications of olanzapine has been confirmed in the literature, and as was confirmed in our previous work [2], the solvent can facilitate the formation of several of them, we analyzed the bottom phases after the dissolution of the solid dispersions. Analysis of the solid residuals after the dissolution in buffer pH 7.4 (Figure S2e) was performed using the diffractograms of the known forms of OLZ from the Cambridge database provided in our previous study [2] and revealed the following. The raw OLZ sample was shown to be a mixture of forms I and II. After the dissolution in pH 7.4 it was identified as Form I. Significant transformation of OLZ towards a poorly crystallized phase in the OLZ/SBE-β-CD (1:1)-SD (Figure S2a) was shown which was transformed to crystalline material mainly corresponding to Form D with small contaminants of forms I, II, IV, and B after the dissolution. For OLZ/GABA SD, the changes after dissolution in the buffer are minor: forms I, II, and B are present in both samples. Only Form IV, which is absent in OLZ/GABA SD, appears after dissolution. Regarding the dissolution of solid dispersions with 6ACA, forms I, II, III, and B are present in the initial sample, whereas after dissolution at pH 7.4, forms III and B remain, and Form D appears.

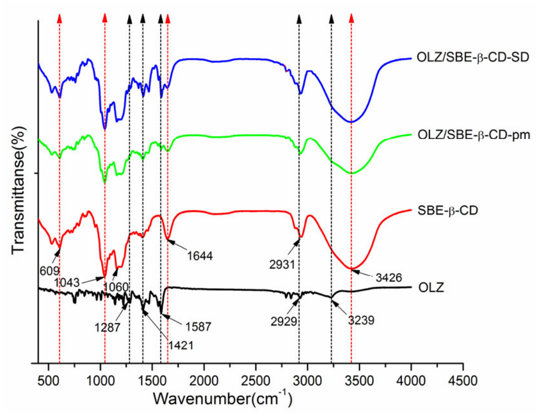

(a)

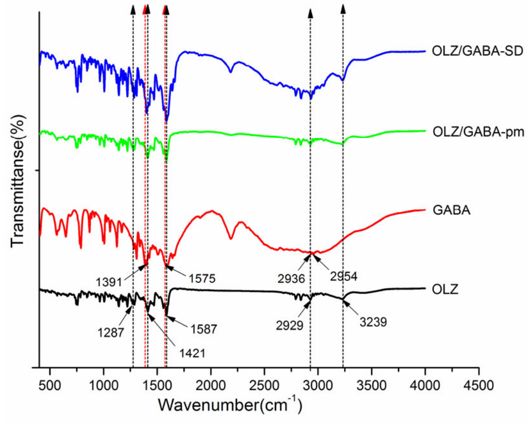

(b)

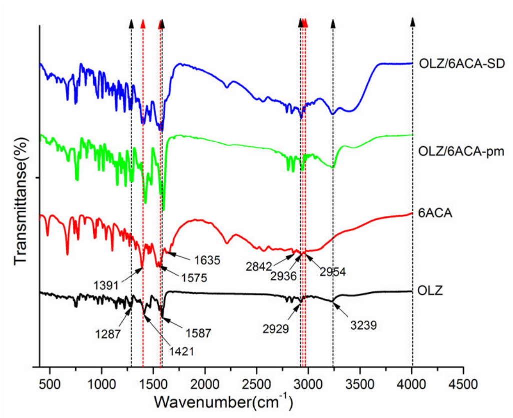

(c)

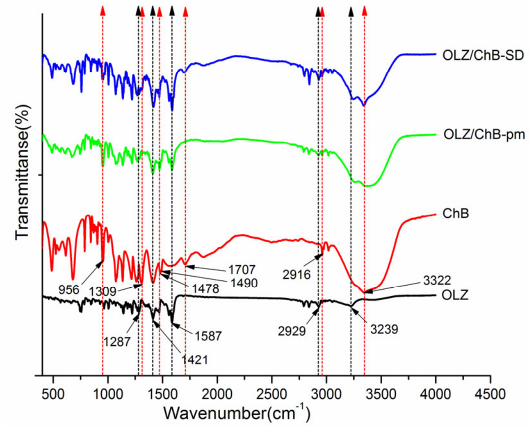

(d)

Figure S3. Infrared spectra of individual compounds: raw OLZ (OLZ), SBE-β-CD (CD), γ-aminobutyric acid (GABA), 6-aminocaproic acid (6ACA), choline bitartrate (ChB); physical mixtures: OLZ/CD-pm, OLZ/GABA-pm, OLZ/6ACA-pm, OLZ/ChB-pm; solid dispersions: OLZ/CD-SD, OLZ/GABA- SD, OLZ/6ACA- SD, OLZ/ChB- SD: (a) systems with SBE-β-CD, (b) systems with GABA, (c) systems with 6ACA, (d) systems with ChB.

Section S4. Description of the IR spectra of individual excipients and two-component systems

The experimental FTIR spectrum of olanzapine showed absorption bands characteristic of specific functional groups: 3239 cm^{-1} (N-H and O-H stretching), 2929 cm^{-1} (C-H stretching), 1587 cm^{-1} (C=C stretching), and 1421 cm^{-1} (C=N stretching), consistent with the literature [3,4]. The FTIR spectrum of SBE-β-CD demonstrated broad bands for O-H stretching at ~3426 cm^{-1} (3700-3000 cm^{-1}) and O-H bending bending at 1644 cm^{-1}. In addition, the absorption band at 2931 cm^{-1} is characteristic of C–H aliphatic stretching vibration, while the peaks at 1160 cm^{-1}, 1043 cm^{-1} and 609 cm^{-1} correspond to glucose unit absorptions. The characteristic OLZ bands at 3239 cm^{-1}, 2929 cm^{-1}, and 1287 cm^{-1} were absent in the spectra of both the pm and SD, whereas the bands at 1587 cm^{-1} and 1421 cm^{-1} remained visible. The bands at 3239 cm^{-1}, 2929 cm^{-1}, and 1287 cm^{-1} have significantly lower intensity compared to the characteristic bands of SBE-β-CD located within a few wavenumbers from them. Consequently, they cannot be visualized and do not confirm an interaction between the components of the SD.

The FTIR spectra of GABA and 6ACA were similar, as shown in (Figs. 3b and 3c, respectively). In accordance with the literature [5] they exhibited characteristic absorption bands at 2954 cm^{-1}, 2936 cm^{-1}, and 2842 cm^{-1}, which are attributed to aliphatic C–H groups. In addition, bands corresponding to the N–H bending at 1635 cm^{-1}, the asymmetrical carboxylate stretching vibration at 1575 cm^{-1}, and the symmetrical carboxylate stretching vibration at 1391 cm^{-1} were also visible. The same bands were observed in the spectrum of

6ACA. However, the bands at 2929 cm^{-1}, 1587 cm^{-1}, and 1421 cm^{-1} overlapped with acid bands at similar wavenumbers.

The principle absorption peaks of ChB (Fig. 3d) were observed at approximately 3322 cm^{-1} (broad O–H stretching), 2916 cm^{-1} (C–H stretching), 1707 cm^{-1} and 956 cm^{-1} (carboxylic acid groups from the bitartrate moiety), 1490 cm^{-1} (CH_2 bending), 1478 cm^{-1} (N–H bending), and 1309 cm^{-1} (C=O stretching). The spectra of the pm and SD forms showed the characteristic broad band of ChB at ~3322 cm^{-1} (which had a reduced width) along with the OLZ band at 3239 cm^{-1}. In addition, the narrow ChB peak at 2916 cm^{-1} disappeared, while the faint OLZ band at 2929 cm^{-1} remained visible. Interestingly, the ChB peaks at 1309 cm^{-1} and 1707 cm^{-1} were detected only in the SD sample.

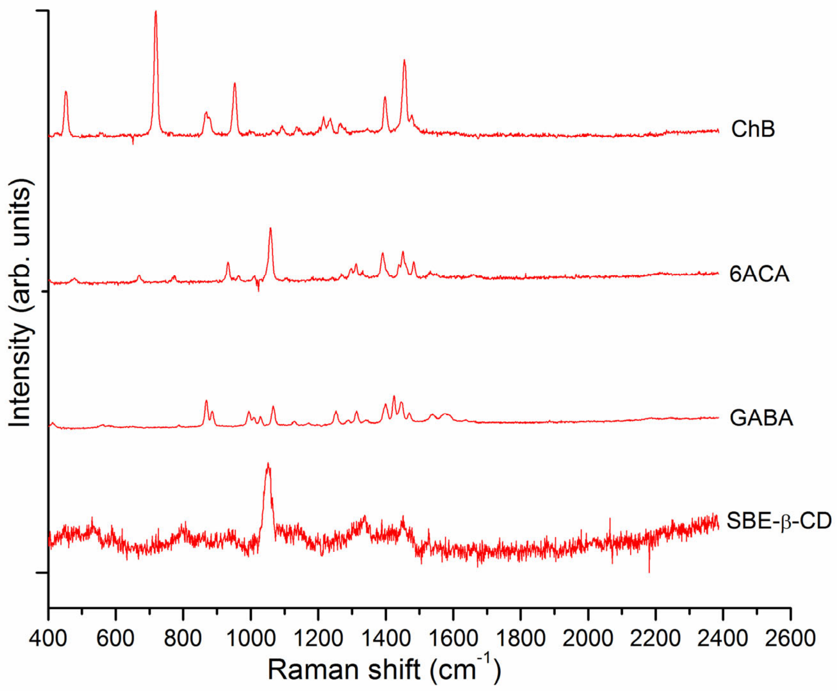

(a)

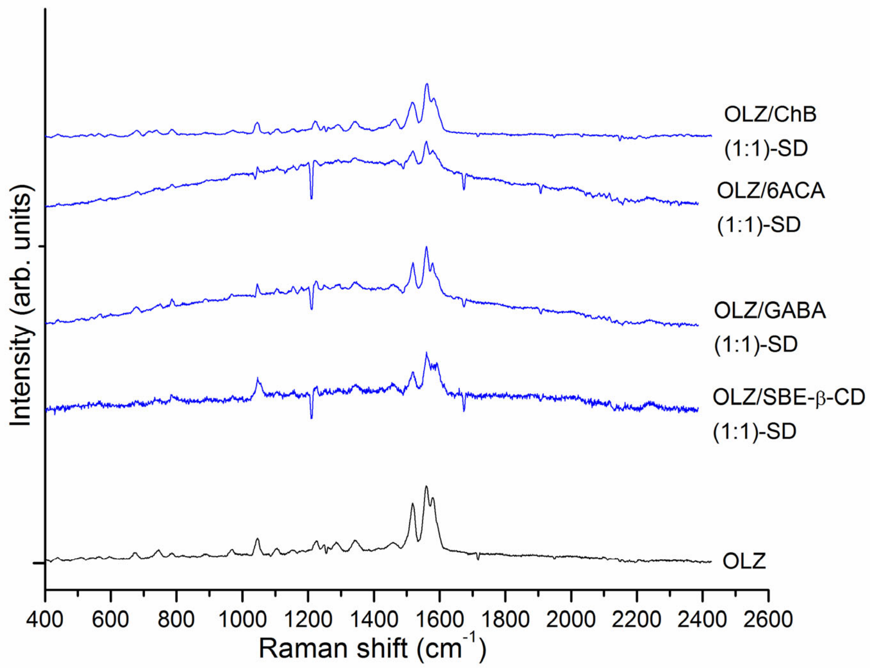

(b)

Figure S4. Raman spectra of excipients: SBE-β-CD (CD), γ-aminobutyric acid (GABA), 6-aminocaproic acid (6ACA), choline bitartrate (ChB) (a), and solid dispersions: OLZ/SBE-β-CD (1:1)-SD, OLZ/GABA (1:1)-SD, OLZ/6ACA (1:1)-SD, OLZ/ChB (1:1)-SD (b).

Section S5. Description of the Raman spectra of individual excipients and two-component systems

The main characteristic bands of OLZ (numbering refers to Fig. 1a) were visible, in agreement with the literature data [3,6,7,8], at the following wavenumbers: 1580 cm^{-1} (R_1, Form II: deformations of the C–H groups in the phenyl rings), 1559 cm^{-1} (R_1, R_2, R_3, Form II: CN and C=C stretching, CH deformation), 1518 cm^{-1} (Form I/Form II: CC stretching, NH and CH_3 deformations), 1457 cm^{-1} (Form I: CH_3 (1) deformations), 1345 cm^{-1} (Form II: R_3 CC stretching, CH deformations; R_4 - CH_2 wagging), 1224 cm^{-1} (Form I: R_1, R_{2b} - ring and CN stretching, CH_2 twisting), 1045 cm^{-1} (Form I/Form II: in-plane breathing deformations of the benzene ring), 965 cm^{-1} (Form I: R_2 and R_4 - ring deformations and twisting, R_3 - CS stretching), 750 cm^{-1} (Form I: R_1 -deformations of the skeleton of the molecule and the lattice vibrations). The presence of these bands in the OLZ spectrum is indicative of its crystallinity, in agreement with previous reports [3]. The Raman spectra of the pure excipients and the prepared two-component SDs are illustrated in Fig. S4a and Fig. S4b, respectively. The spectral region between 1600 cm^{-1} and 1500 cm^{-1} was attributed to double-bond vibrations

partially coupled to CH and NH bending deformations (from the benzene and thiophene rings, and the CN bond of the azepine ring). These vibrations are strongly enhanced in Raman spectroscopy and are often weak or absent in FTIR spectra [7].

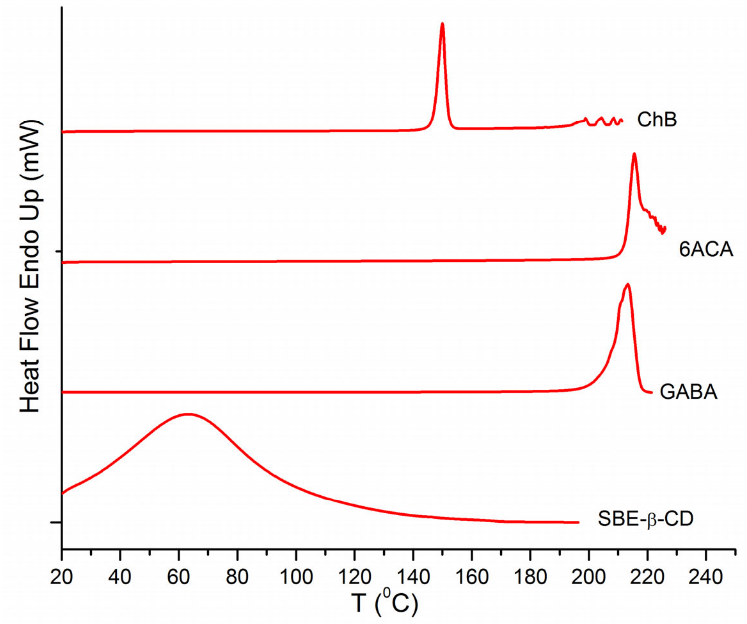

(a)

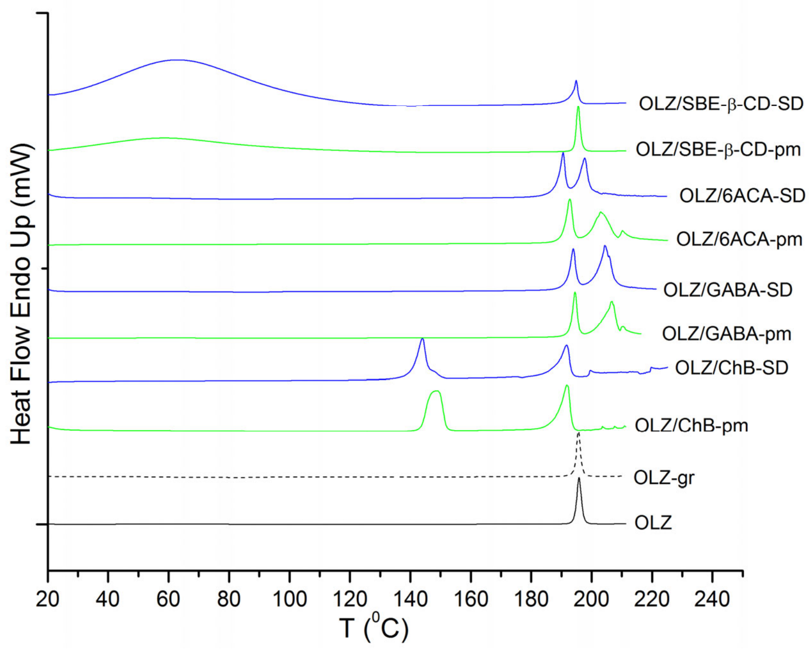

(b)

Figure S5. DSC patterns of excipients: SBE-β-CD (CD), γ-aminobutyric acid (GABA), 6-aminocaproic acid (6ACA), choline bitartrate (ChB) (a), two-component solid samples: physical mixtures (pm) and solid dispersions (SD) with OLZ (b).

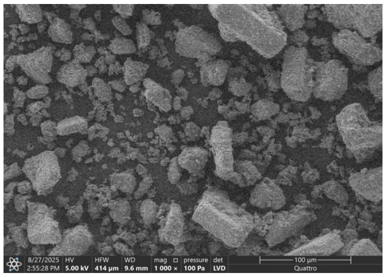
(a) 50 µm/30 µm

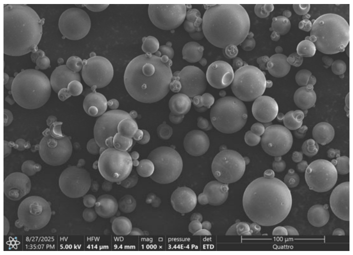
(b) 50 µm/30 µm

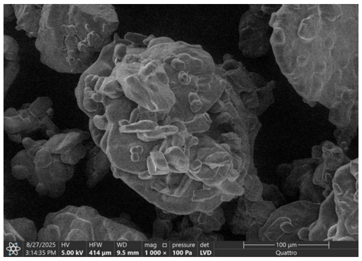
(c) 200 µm/100 µm

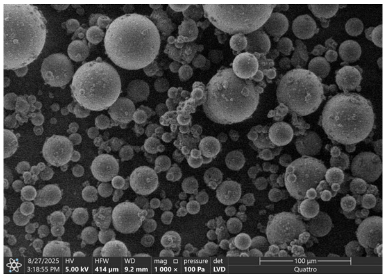
(d) 50 µm/30 µm

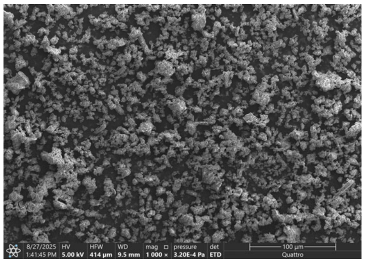
(e) indefinite sizes

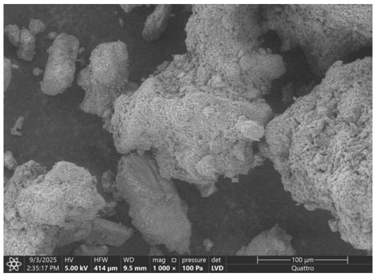
(f) 200 µm/75 µm

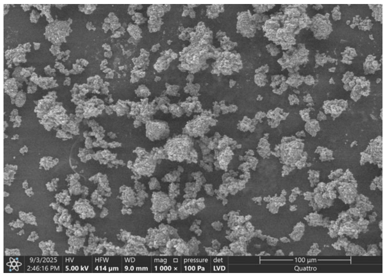
(g) 30 µm/20 µm

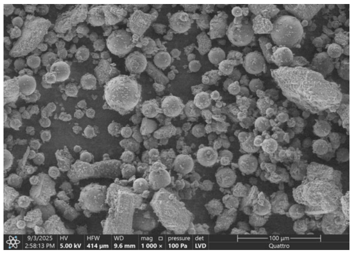
(h) 40 µm/20 µm

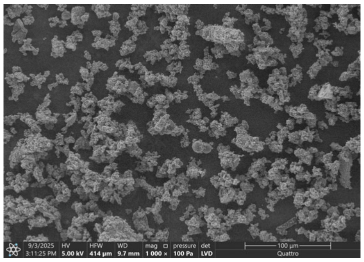
(i) indefinite sizes

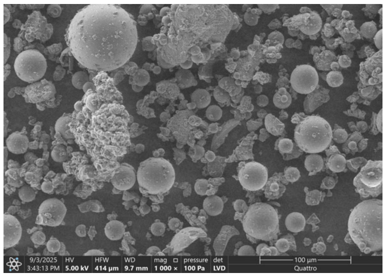
(j) 150 µm/50 µm

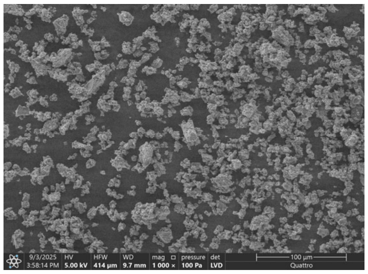
(k) indefinite sizes

Figure S6. SEM micrographs of raw OLZ (a), SBE-β-CD (b), GABA (c), OLZ/SBE-β-CD (1:1)-pm (d), OLZ/SBE-β-CD (1:1)-SD (e), OLZ/GABA (1:1)-pm (f), OLZ/GABA (1:1)-SD (g), OLZ/SBE-β-CD/GABA (1:0.25:0.75)-pm (h), OLZ/SBE-β-CD/GABA (1:0.25:0.75)-SD (i), OLZ/SBE-β-CD/GABA (1:0.75:0.25)-pm (j), OLZ/SBE-β-CD/GABA (1:0.75:0.25)-SD (k). Applied magnification was (×1000). The maximal sizes/average sizes of the particles where applicable are indicated just below the respective images.

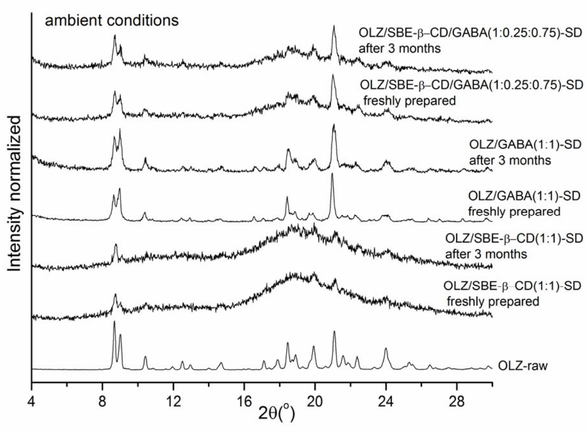
ambient conditions
OLZ/SBE-β-CD/GABA(1:0.25:0.75)-SD after 3 months
OLZ/SBE-β-CD/GABA(1:0.25:0.75)-SD freshly prepared
OLZ/GABA(1:1)-SD after 3 months
OLZ/GABA(1:1)-SD freshly prepared
OLZ/SBE-β-CD(1:1)-SD after 3 months
OLZ/SBE-β-CD(1:1)-SD freshly prepared
OLZ-raw
Intensity normalized
2θ(°)
4
8
12
16
20
24
28

(a)

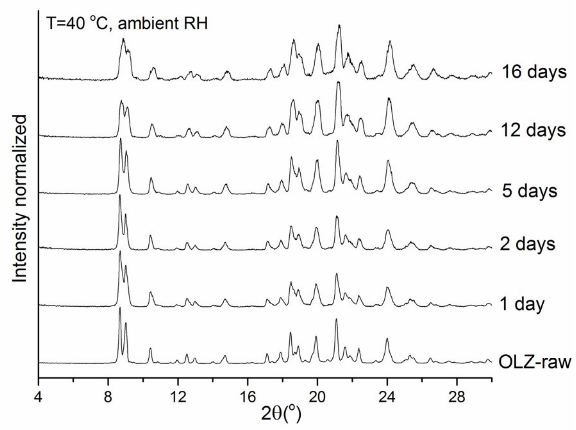
T=40 °C, ambient RH
16 days
12 days
5 days
2 days
1 day
OLZ-raw
Intensity normalized
2θ(°)
4
8
12
16
20
24
28

(b)

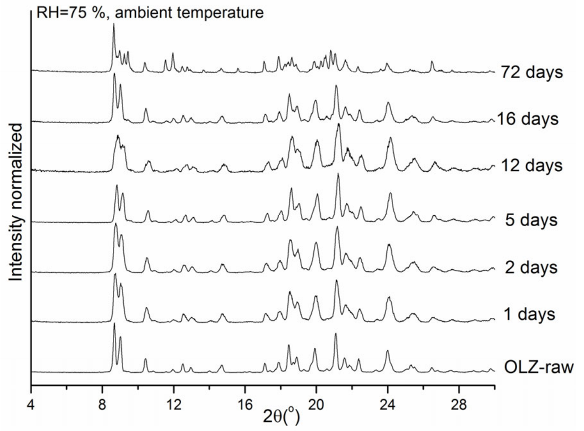
RH=75 %, ambient temperature
72 days
16 days
12 days
5 days
2 days
1 days
OLZ-raw
Intensity normalized
2θ(°)
4
8
12
16
20
24
28

(c)

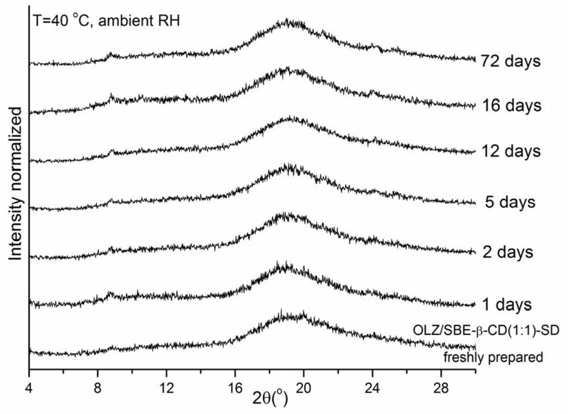
T=40 °C, ambient RH
72 days
16 days
12 days
5 days
2 days
1 days
OLZ/SBE-β-CD(1:1)-SD freshly prepared
Intensity normalized
2θ(°)
4
8
12
16
20
24
28

(d)

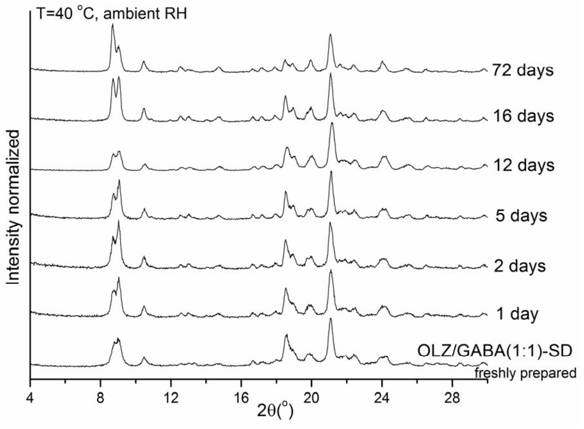
T=40 °C, ambient RH
72 days
16 days
12 days
5 days
2 days
1 day
OLZ/GABA(1:1)-SD freshly prepared
Intensity normalized
2θ(°)
4
8
12
16
20
24
28

(e)

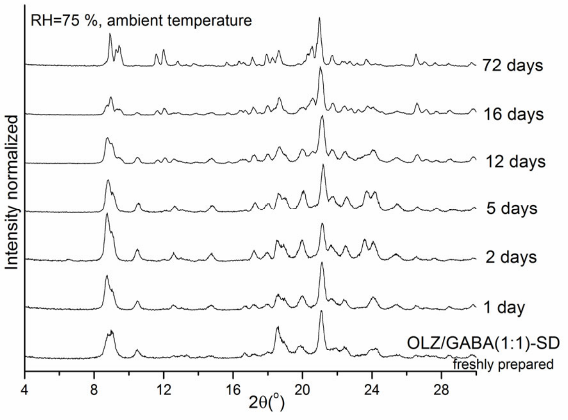
RH=75 %, ambient temperature
72 days
16 days
12 days
5 days
2 days
1 day
OLZ/GABA(1:1)-SD freshly prepared
Intensity normalized
2θ(°)
4
8
12
16
20
24
28

(f)

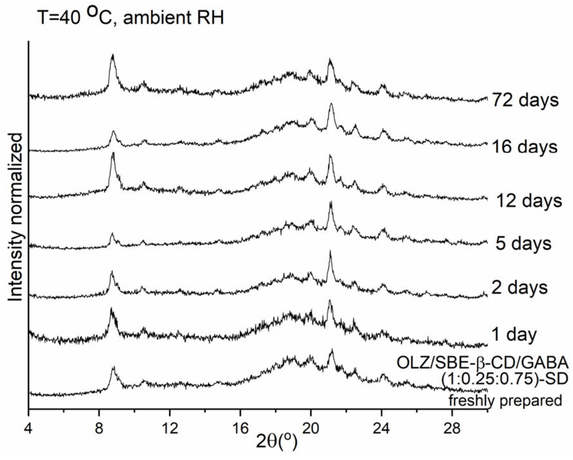

(g)

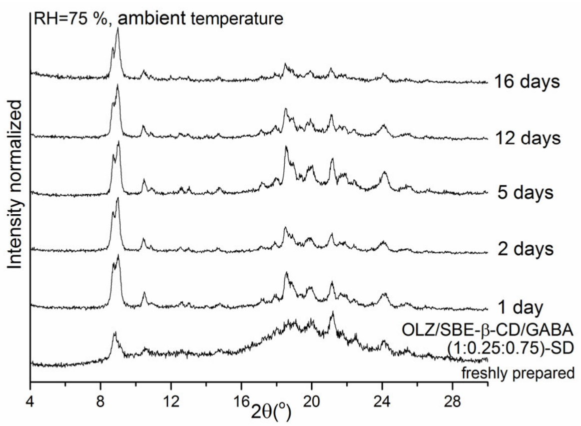

(h)

Figure S7. Stability at different storage conditions: selected SDs at ambient conditions (a), OLZ, T=40 ºC/ambient RH (b), OLZ, RH=75 %/ambient temperature (c), OLZ/SBE-β-CD (1:1), T=40 ºC/ambient RH (d), OLZ/GABA (1:1), T=40 ºC/ambient RH (e), OLZ/GABA (1:1), RH=75 %/ambient temperature (f), OLZ/SBE-β-CD/GABA (1:0.25:0.75)-SD, T=40 ºC/ambient RH (g), OLZ/SBE-β-CD/GABA (1:0.25:0.75)-SD, RH=75 %/ambient temperature (h).

Table S1. Sample Table.

Compound Name	Abbreviation	Formula	Molecular weight (g·mol^{-1})	CAS No.	Supplier	Purity (%)
Olanzapine	OLZ	$C_{17}H_{20}N_4S$	312.44	132539-06-1	Aladdin Scientific	≥99
Sulfobutylether-β-cyclodextrin sodium salt (Captisol)	SBE-β-CD	$C_{50}H_{84}Na_2O_{41}S_2$	1451.3	182410-00-0	BLDpharm	99
Choline bitartrate	ChB	$C_9H_{19}NO_7$	253.25	87-67-2	Shanghai Macklin Biochemical Co. Ltd.	98
γ-Aminobutyric acid	GABA	$C_4H_9NO_2$	103.12	56-12-2	Sigma	≥99
6-Aminocaproic acid	6ACA	$C_6H_{13}NO_2$	131.175	60-32-2	Sigma	≥99
Potassium dihydrogen phosphate	KH_2PO_4	KH_2PO_4	136.086	7778–77-0	Merck	≥99
Disodium hydrogen phosphate dodecahydrate	$Na_2HPO_4\cdot 12H_2O$	$Na_2HPO_4\cdot 12H_2O$	358.14	10039–32-4	Merck	≥99

References

1. Sarge, S.M.; Hemminger, W.; Gmelin, E.; Hohne, G.W.H.; Cammenga, H.K.; Eysel, W. Metrologically based procedures for the temperature, heat and heat flow rate calibration of DSC. *J. Therm. Anal.* **1997**, *49*, 1125–1134. https://doi.org/10.1007/BF01996802.
2. Volkova, T.V.; Simonova, O.R.; Perlovich, G.L. Modulation of solubility, distribution and permeability of olanzapine: Selection of pharmaceutical excipients and mechanistic investigation. *Colloids Surf. A* **2026**, *728*, 138728. https://doi.org/10.1016/j.colsurfa.2025.138728.
3. Krishnamoorthy, V.; Suchandrasen; Prasad, V.P.R. Physicochemical characterization and in vitro dissolution behavior of olanzapine-mannitol solid dispersions. *Brazilian J. Pharm. Sci.* **2012**, *48*(2), 243–255. https://doi.org/10.1590/S1984-82502012000200008.
4. Hiriyanna, S.G.; Basavaiah, K.; Goud, P.S.K.; Dhayanidhii, V.; Raju, K.; Patil, H.N. Identification and characterization of olanzapine degradation products under oxidative stress conditions. *Acta Chromatogr*. **2008**, *20*, 81–93. https://doi.org/10.1556/AChrom.20.2008.1.7.
5. Zareie, Z.; Tabatabaei Yazdi, F.; Mortazavi, S.A. Optimization of gamma-aminobutyric acid production in a model system containing soy protein and inulin by Lactobacillus brevis fermentation. *Food Measure* **2019**, *13*, 2626–2636. https://doi.org/10.1007/s11694-019-00183-8.
6. Bhardwaj, R.M. Exploring the crystal structure landscape of olanzapine, In book: Control and Prediction of Solid-State of Pharmaceuticals, Chapter 6, pp. 99−151. https://doi.org/10.1007/978-3-319-27555-0_6.
7. Ayala, A.P.; Siesler, H.W.; Boese, R.; Hoffmann, G.G.; Polla, G.I.; Vega, D.R. Solid state characterization of olanzapine polymorphs using vibrational spectroscopy. *Int. J. Pharm*. **2006**, *326*, 69–79. https://doi.org/10.1016/j.ijpharm.2006.07.023.
8. Polla, G.I.; Vega, D.R.; Lanza, H.; Tombari, D.G.; Baggio, R.; Ayala, A.P.; Filho, J.M.; Fernandez, D.; Leyva, G.; Dartayet, G. Thermal behaviour and stability in Olanzapine. *Int. J. Pharm*. **2025**, *301*, 33–40. https://doi.org/10.1016/j.ijpharm.2005.05.035.
